# A Quick Access to Structurally Diverse Triazoloquinazoline Heterocycles *via* the MIL-101(Cr)-Catalyzed One-Pot Multi-Component Reaction of a Series of Benzaldehydes, Dimedone, and 1*H*-1,2,4-Triazol-3-Amine Under Green Conditions

**DOI:** 10.3389/fchem.2022.898658

**Published:** 2022-07-25

**Authors:** Pezhman Shiri, Esmaeil Niknam, Jasem Aboonajmi, Ali Khalafi-Nezhad, Ali Mohammad Amani

**Affiliations:** ^1^ Department of Medical Nanotechnology, School of Advanced Medical Sciences and Technologies, Shiraz University of Medical Sciences, Shiraz, Iran; ^2^ Department of Medical Biotechnology, School of Advanced Medical Sciences and Technologies, Shiraz University of Medical Sciences, Shiraz, Iran; ^3^ Department of Chemistry, College of Sciences, Shiraz University, Shiraz, Iran

**Keywords:** short reaction times, green conditions, heterogeneous catalyst, MIL-101(Cr), heterocycles

## Abstract

A one-pot multicomponent reaction of a variety of benzaldehydes, dimedone, and 1*H*-1,2,4-triazol-3-amine for the efficient synthesis of quinazolinone derivatives under green conditions is reported. It was proved that MIL-101(Cr) could carry out successfully this multicomponent strategy to afford target products in high yields. The scope and limitation of this catalytic system concerning the aldehyde substrates were explored. Different aldehydes could be conveniently delivered to quinazolinones at room temperature with short reaction times in an atom-economy way. Notably, MIL-101(Cr) was also characterized by different analytic methods such as FT-IR, SEM, and EDX. The outstanding benefits of this methodology are the availability of substrates, using green conditions, excellent functional group compatibility, and reusability of catalysts, therefore providing easy access to a range of products of interest in organic and medicinal chemistry.

## Introduction

Nitrogen-containing heterocycles occur in natural products (Yadav et al., 2014), ionic liquids (Wang et al., 2006), bioactive molecules (Kundu et al., 1999; Wiglenda et al., 2005; Wiglenda and Gust, 2007; Mashayekh and Shiri, 2019), and dyes (Wang et al., 2013). A concise review of the most active molecules displays that *N*-heterocyclic systems are the most dominant scaffolds of biologically active molecules (Alizadeh et al., 2017a; Alizadeh et al., 2017b; Sharghi et al., 2018; Alizadeh et al., 2019; Mashayekh and Shiri, 2019; Henary et al., 2020; Shiri, 2020). They are of much interest as antitumor agents (Al-Soud et al., 2003; Shiri, 2021), antibacterial agents (Dixit et al., 2005; Shiri and Aboonajmi, 2020), antipsychotic agents (Perregaard et al., 1992), antiestrogens (Von Angerer et al., 1987), cyclooxygenases (COX)-1 inhibitors (Sano et al., 2006), and antifungal agents (Rezaei et al., 2009) and also have wide usage in materials science (Novak et al., 2009; Anderson and Long, 2010).

Quinazolinone derivatives have been proven to exhibit interesting medicinal and therapeutical activities, including antitumor, antihypertensive, antifungal, analgesic and anti-inflammatory, antibacterial, antihistaminic, anticancer, antioxidant, and anti-HIV activities (Karan et al., 2021). To the best of our knowledge, a few reports are available for the preparation of triazoloquinazolinones by condensation of 3,5-diamino-1,2,4-triazole, aromatic aldehydes, and dimedone (Lipson et al., 2003; Heravi et al., 2008; Heravi et al., 2010; Krishnamurthy and Jagannath, 2013; Puligoundla et al., 2013; Mousavi and Maghsoodlou, 2015) such as H_6_P_2_W_18_O_62_•18H_2_O (Heravi et al., 2008), microwave (Krishnamurthy and Jagannath, 2013), molecular iodine (I_2_) (Puligoundla et al., 2013), refluxing in DMF (Lipson et al., 2003), and more recently, TiO_2_ nanoparticles supported ionic liquids (Bakhshali-Dehkordi et al., 2020), Sc(OTf)_3_ (Gajaganti et al., 2018) and DABCO-based ionic liquid supported on Fe_3_O_4_@TiO_2_ nanoparticles (Bakhshali‐Dehkordi et al., 2020). Despite the usefulness of these catalysts, they also have some limitations, including high toxicity, use of expensive materials, complex synthetic processes, requirement of expensive and hazardous catalysts, and sometimes low yields.

Performing the transformation in one-pot multicomponent has gained much attention in the view point of environmental friendliness, energy efficiency, and atom economy (Aboonajmi et al., 2020; Keri et al., 2021; Shiri and Amani, 2021; Shiri et al., 2021). Nowadays, multicomponent strategies have a superior position in the organic and pharmacy industries. The concept of multicomponent refers to combining more than two substrates in a one-pot reaction, providing more complexity and diversity in a green way. Indeed, the significance of this subject can be highlighted by the considerable number of related articles and review articles (Khangah et al., 2021; Bedard et al., 2022; Liu et al., 2022). Moreover, the construction of numerous compounds that are crucial to address the necessities of our societies must be handled with key terms such as synthetic efficiency and, more importantly, atom economy by increasing the utilization of the number of atoms in the substrates that end up in the structure of the final products (Biesen and Müller, 2021).

In recent years, tremendous development has been achieved in the field of MOFs (metal−organic frameworks) architectures as a novel class of porous materials. The MOFs incorporate the advantages of sustainable synthesis with heterogeneous catalysis, which indeed make easier workup procedure, and are more applicable in industries and academics. Due to global energy and environmental problems, the finding of unique catalysts in a simple way with high reusability has become increasingly crucial in managing the associated challenges. An increase in the number of articles and review articles on the subject of MOFs as novel heterogeneous catalysts has demonstrated a profound interest in investigating these structures and their applications as a new class of catalysts (Koolivand et al., 2021; Ma et al., 2021).

In the light of these considerations, based on our previous research, which focused on the heterocyclic systems under green conditions (Shiri et al., 2021), we aimed to report our efforts to carry out the one-pot multicomponent synthesis of structurally diverse quinazolinone heterocycles using a novel, efficient, and easily prepared MIL-101(Cr) catalyst in the current study.

## Experimental Section

### Instrumentation, Analyses, and Starting Materials

Chemical materials and solvents were either synthesized in our laboratory or purchased from Fluka, Aldrich, and Merck Companies. NMR spectra were recorded on a Bruker Avance DPX-250 (^1^H-NMR 250 MHz and ^13^C-NMR 62.9 MHz) spectrometer in pure deuterated solvents with tetramethylsilane as an internal standard. The purity determination of the starting materials and monitoring of reactions were accomplished by TLC on silica gel PolyGram SILG/UV 254 plates.

### Synthetic Route for the MIL-101(Cr) Catalyst

MIL-101(Cr) catalyst was synthesized according to the previously reported procedure (Niknam et al., 2018). First, a mixture of Cr(NO_3_)_3_·9H_2_O (5.4 g) and terephthalic acid (1.5 g) was added to deionized water (45 ml) and hydrofluoric acid (0.6 ml, 5 mol L^−1^) in a Teflon-lined stainless steel autoclave. After sonication for 10 min, the mixture was heated in an oven at 220°C for 9 h. In continuation, the mixture was cooled down to r. t., and then the mixture was filtered and washed several times with hot water and hot DMF. The resulting MIL-101(Cr) catalyst was dried and purified further with hot filtration in DMF at 120°C for 12 h. The solid was washed several times with hot DMF and hot ethanol. Finally, the solid was filtered and dried at 80°C for 6 h. MIL-101(Cr) catalyst was characterized, as mentioned in the Results and Discussion section (Niknam et al., 2018).

### General Procedure for the One-Pot Multicomponent Synthesis of Structurally Diverse Quinazolinone Heterocycles

MIL-101(Cr) (7.0 mg) as the catalyst was added to a mixture of aldehyde (1.0 mmol), dimedone (1.0 mmol), and 1*H*-1,2,4-triazol-3-amine (1.0 mmol) in acetonitrile (2.0 ml) and stirred at r. t. for an appropriate time. After the completion of the reaction, the catalyst was separated by centrifugation which was used for the next run. After evaporation of the solvent, the product was obtained using recrystallization of the solid residue in hot ethanol.

## Results and Discussion

### Synthesis and Characterization of MIL-101(Cr)

**FIGURE 1:**
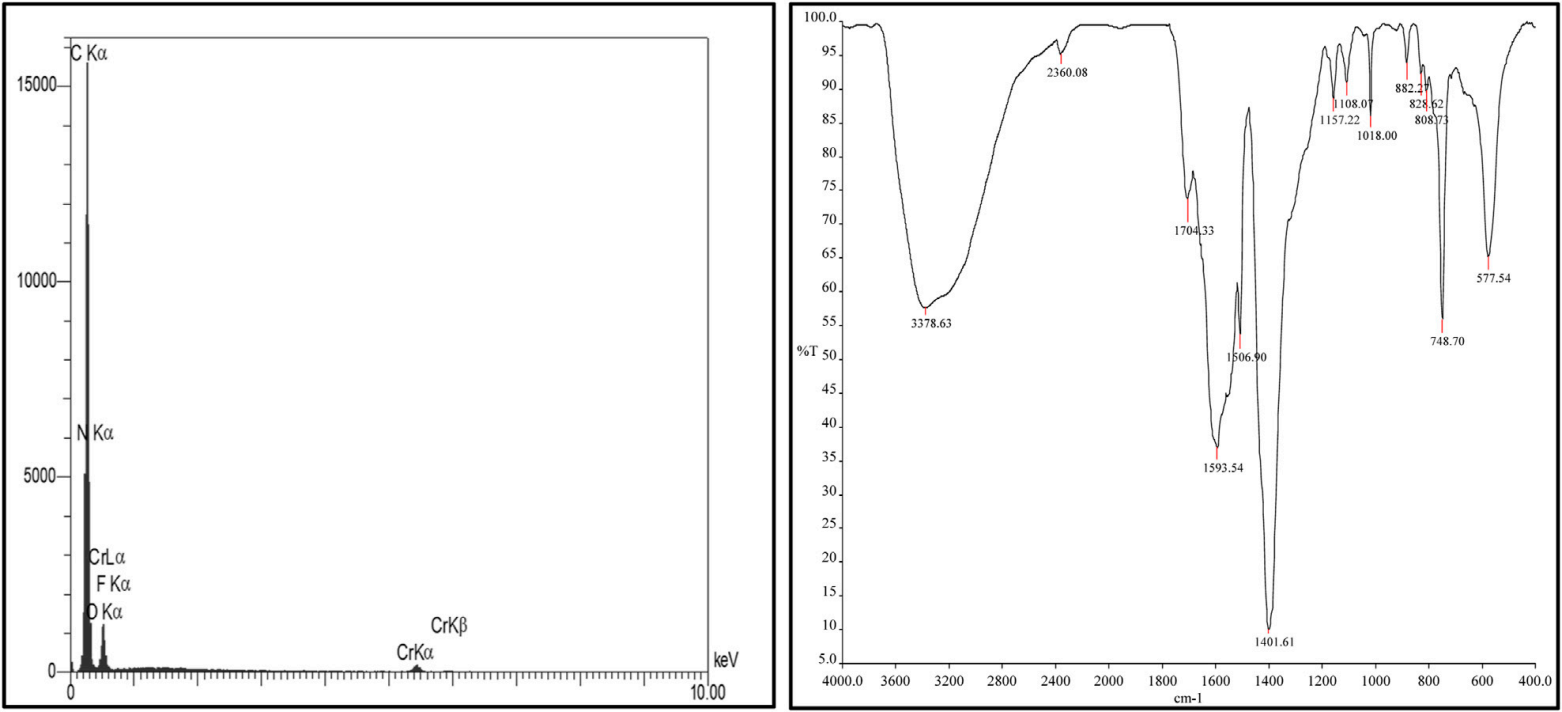
EDX analysis and the FT-IR spectrum of the MIL-101(Cr) nanocatalyst.

First, MIL-101(Cr) nanocatalyst was characterized using FT-IR spectrum displaying index peaks in agreement with the literature (Figure 1). Figure 1 shows the EDX spectrum of MIL-101(Cr) nanocatalyst. This spectrum proved the elements of C, O, Cr, F, and N in the composition (Niknam et al., 2018; Yang and Yan, 2011).

**FIGURE 2:**
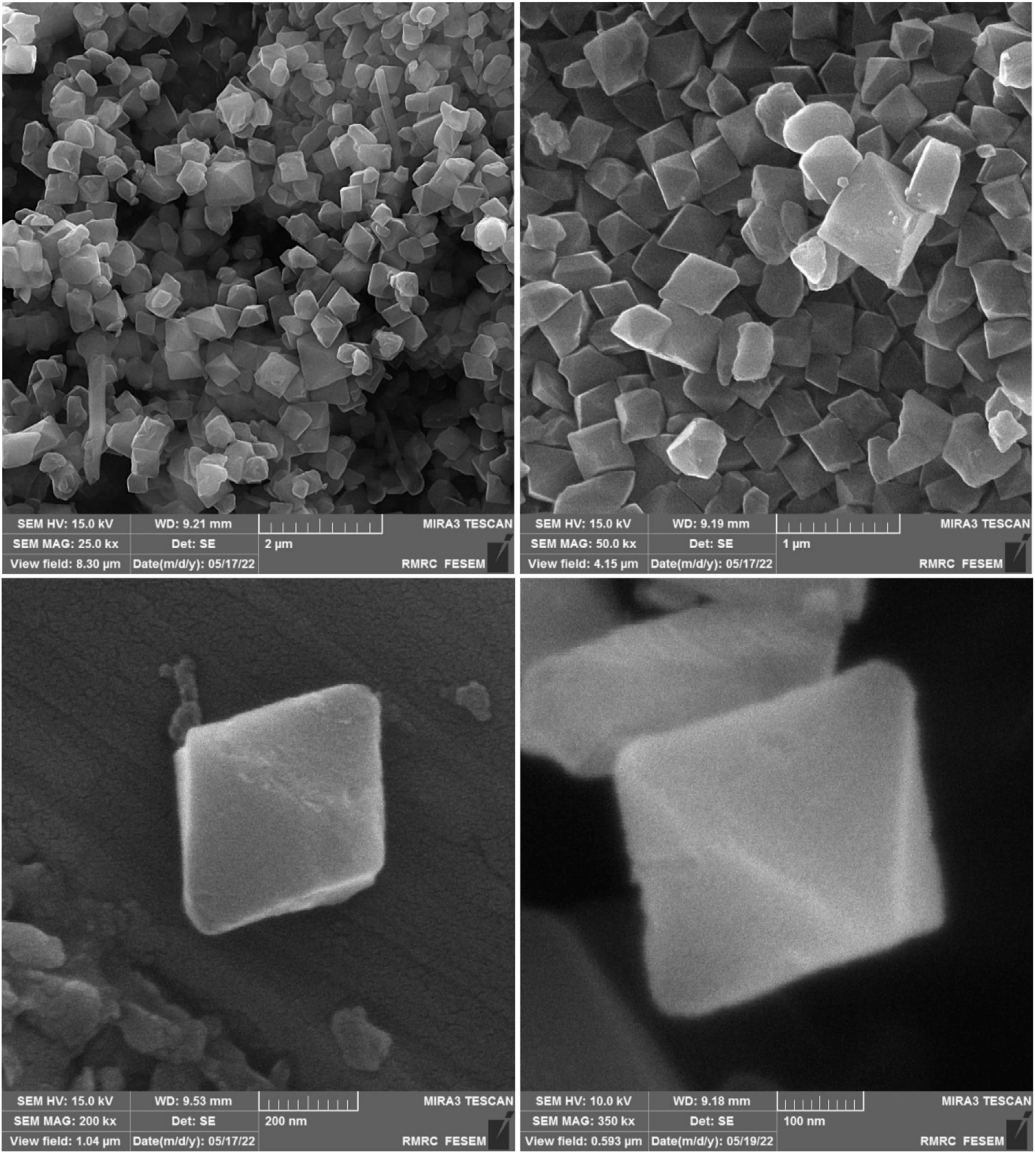
SEM images of the MIL-101(Cr) nanocatalyst.

The SEM images of the MIL-101(Cr) nanocatalyst also exhibited discrete octahedrons containing smooth surfaces with an average size of 350 nm. The SEM images also displayed octahedron MIL-101(Cr) nanocatalyst, but they were not uniform (Figure 2). The content of Cr in the synthesized nanocatalysts was determined by ICP to be about 0.35 mmol g^−1^ of MIL-101(Cr) (Niknam et al., 2018).

### Application of MIL-101(Cr) as a Novel Heterogeneous Catalyst for the One-Pot Multicomponent Synthesis of Structurally Diverse Quinazolinone Heterocycles

For our primary exploring experiments, 1*H*-1,2,4-triazol-3-amine (**1**), dimedone (**3**), and benzaldehyde (**2a**) were chosen as the model starting materials to optimize the reaction conditions. Initially, the effect of several solvents on the coupling of 1*H*-1,2,4-triazol-3-amine (**1**), dimedone (**3**), and benzaldehyde (**2a**) was explored in the presence of MIL-101(Cr) as a novel heterogeneous catalyst (7 mg) at r. t. (Table 1, entries 1–5). The usage of acetonitrile as solvent gave the corresponding product **4a** in 94% yield (Table 1, entries 1), whereas other solvents, including methanol, ethanol, water, and ethanol/water were considerably less effective and provided lower yields of the product (Table 1, entries 2–5). Acetonitrile was found to be the most effective solvent, affording quinazolinone heterocycles **4a** with a yield of 94% (Table 1, entry 1).

**TABLE 1:** Optimization of the reaction conditions for the one-pot multicomponent synthesis of quinazolinone **4a** from 1*H*-1,2,4-triazol-3-amine (**1**), benzaldehyde (**2a**), and dimedone (**3**)^a^ 
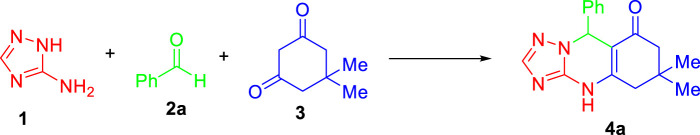
.

Entry	Catalyst loading	Solvent	Temp. (°C)	Time (min)	Yield (%)	TON^b^	TOF(h^−1^)^c^
1	7 mg (0.25 mol%)	acetonitrile (4 ml)	r.t.	30	94	376	752
2	7 mg (0.25 mol%)	ethanol (4 ml)	r.t.	45	60	240	320
3	7 mg (0.25 mol%)	methanol (4 ml)	r.t.	45	50	200	266.7
4	7 mg (0.25 mol%)	water (4 ml)	r.t.	45	45	180	240
5	7 mg (0.25 mol%)	water/ethanol (4 ml)	r.t.	45	52	208	277.4
6	3.5 mg (0.12 mol%)	acetonitrile (4 ml)	r.t.	30 (60)	81 (89)	675 (741.7)	1350 (741.7)
7	9 mg (0.32 mol%)	acetonitrile (4 ml)	r.t.	30	93	290.6	581.2
8	-	acetonitrile (4 ml)	r.t.	45	Trace	-	-
**9**	**7 mg** (0.25 mol%)	**acetonitrile (2 ml)**	**r.t.**	**30**	**94**	**376**	**752**
10	7 mg (0.25 mol%)	acetonitrile (4 ml)	reflux	15	93	372	1488

a The reactions were carried out with of 1*H*-1,2,4-triazol-3-amine (**1**, 1.0 mmol), benzaldehyde (**2a**, 1.0 mmol), and dimedone (**3**, 1.0 mmol).

b TON = mmol product/mol% catalyst.

c TOF = TON/reaction time (h^−1^).

In the next step, the alteration of catalyst loading was explored for the coupling reaction. The highest yield (94%) of the product was formed when 7 mg (0.25 mol%) of the catalyst was exploited (Table 1, entries 1 and 9). By decreasing the catalyst loading to 3.5 mg (0.12 mol%), the product was achieved in 81% yield (Table 1, entry 6), whereas it was observed that increasing the catalyst loading had no significant effect on the product yield (Table 1, entry 7). Moreover, without using MIL-101(Cr), the reaction failed to proceed even after 45 min (Table 1, entry 8). The reaction time was sharply decreased to 10 min once the reaction was performed under reflux conditions (Table 1, entry 10).

Finally, for decreasing the level of environmental pollution, the volume of used solvent was decreased to 2 ml (Table 1, entry 9). It was observed that **4a** was achieved in 94% yield in the presence of MIL-101(Cr) (7 mg) in acetonitrile (2 ml) at r. t. for 30 min (Table 1, entry 9).

Next, the scope and limitations of this catalytic system were explored with respect to aldehyde substrates (**2**). A wide range of aldehyde compounds (**2**) was coupled with 1*H*-1,2,4-triazol-3-amine (**1**) and dimedone (**3**) under standard reaction conditions to form excellent yields of desired quinazolinone derivatives (**4**). The results of this exploration are illustrated in Table 2. Diverse aldehyde compounds (**2a-m**) containing electron-donating or electron-deficient functional groups were coupled with their partners to produce high yields of corresponding quinazolinones (**4a-m**).

**TABLE 2:** Synthesis of a variety of quinazolinones under optimized conditions^a^ ^b^.

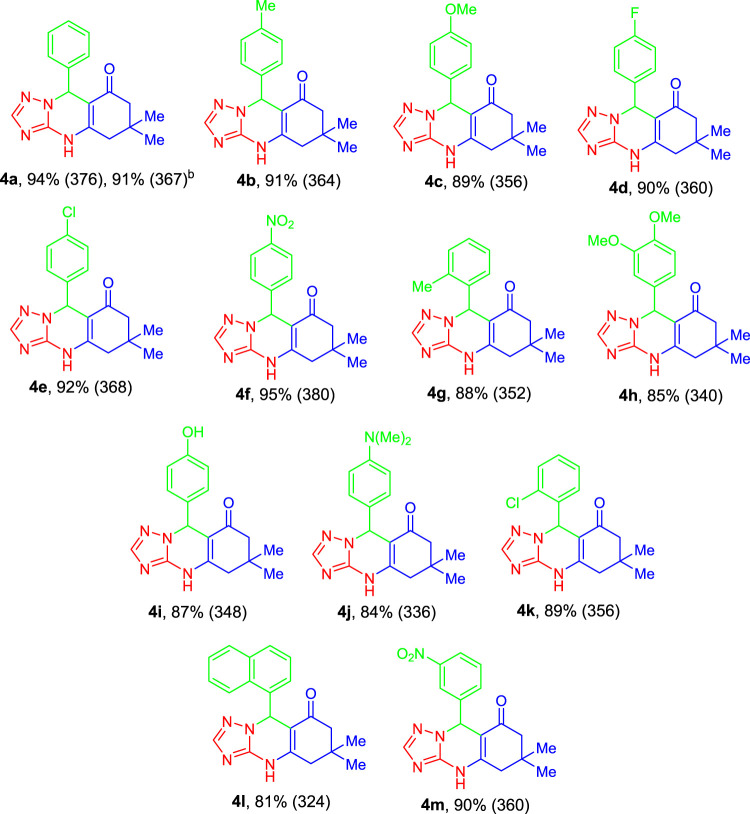

a Coupling of 1*H*-1,2,4-triazol-3-amine (**1**, 1.0 mmol), dimedone (**2**, 1.0 mmol), and benzaldehyde derivatives (**3**, 1.0 mmol) has been performed under standard conditions for 30 min.

b The numbers in the parentheses are TON.

c The yield for a 10 mmol scale.

It is worth noting that the corresponding products could be obtained with both electron-releasing and electron-withdrawing groups on different positions of arenes such as 4-OH, 3-NO_2_, 4-Me_2_N, 2-Cl, 3,4-diMeO, 2-Me, 4-Me, 4-Cl, 4-MeO, 4-NO_2,_ and 4-F in high yields within a short reaction time (Table 2). The study displayed that there is no notable difference between these functional groups except in the case of ortho-substituted aldehyde substrates, which could be due to steric hindrance. Naphthaldehyde could also tolerate the reaction conditions to afford the target product in 81% yield. The reaction for the synthesis of quinazolinone could be scaled up under the standard reaction conditions without a significant decrease in the yield of **4a** (Table 2).

**SCHEME 1:**
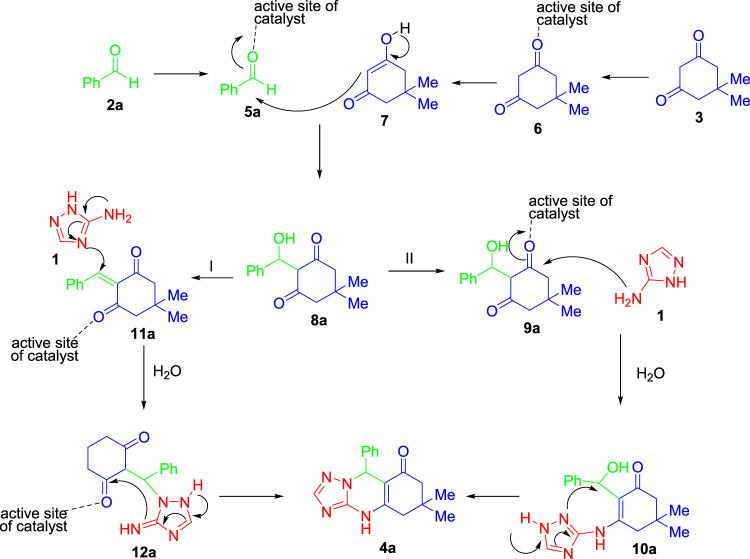
Mechanism for the coupling reaction between 1*H*-1,2,4-triazol-3-amine (**1**), benzaldehyde (**2a**), and dimedone (**3**) in the presence of MIL-101(Cr) as a novel catalyst.

**FIGURE 3:**
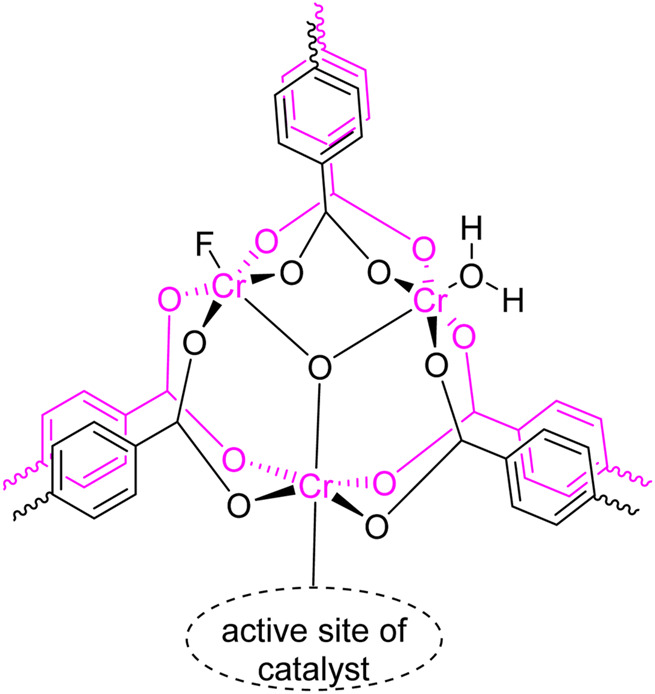
Proposed structure for the active site of the MIL-101(Cr) catalyst.

Based on literature studies (Bakhshali-Dehkordi et al., 2020), herein, we assumed a possible mechanism for the one-pot multicomponent reaction of 1*H*-1,2,4-triazol-3-amine (**1**), benzaldehyde (**2a**), and dimedone (**3**) in the presence of MIL-101(Cr) as a novel catalyst (Scheme 1). According to the structure of MIL-101(Cr) reported in the literature, the active catalytic site of MIL-101(Cr) is conceivable, as shown in Figure 3 (Niknam et al., 2018). The reaction could proceed *via* two reasonable mechanisms to afford the corresponding product (**4a**) in the presence of MIL-101(Cr) as a catalyst. According to the proposed route I, the reaction may carry out through the coupling of **7** and **5a** (named Knoevenagel condensation) to give *α*,*β*-unsaturated carbonyl compounds **8a** with the loss of a water molecule. In the next step, an intermolecular Mannich-type reaction followed by annulation transformation may produce the final product **4a**. The reaction may also give the final product **4a** *via* route II. According to this route, 8a is activated by MIL-101(Cr) as a catalyst for further nucleic attack of amine substrate (**1**). Upon realizing two water molecules and intramolecular annulation, final product **4a** is obtained (Scheme 1).

**FIGURE 4:**
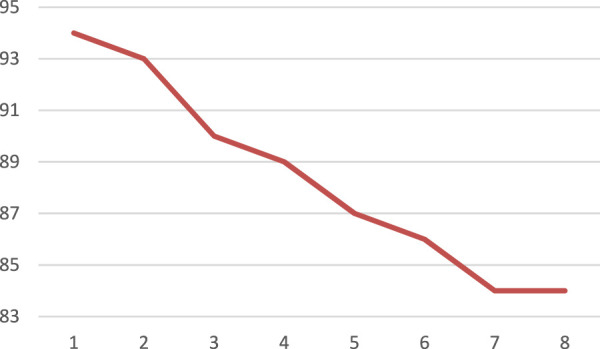
Investigation of reusability of the MIL-101(Cr) catalyst within eight runs.

The stability and recovery of catalysts are crucial aspects for modern societies challenged with economic and environmental issues. In this regard, screening of the reusability of MIL-101(Cr) as a novel catalyst must be performed. Therefore, the reusability of the catalyst was explored for the model reaction. In order to reuse MIL-101(Cr) as a novel catalyst, the mixture of reaction was centrifuged to separate the MIL-101(Cr) catalyst, and then the MIL-101(Cr) catalyst was washed several times with DCM. After drying, the MIL-101(Cr) catalyst was utilized for the next run. To our delight, the MIL-101(Cr) catalyst could be used for eight runs (Figure 4). The major issue in the case of heterogeneous catalysis is the leaching of active centers during the reaction. We tested the leaching experiment (heterogeneity test) of this catalyst. The evaluation of the Cr content in the structure of the catalyst utilizing ICP analysis revealed that about 6.7% of Cr was removed from the MIL-101(Cr). The reused catalyst from the reaction mixture was also characterized by IR analysis, and the results are shown in Supplementary Scheme S1.

A comparative analysis of catalytic activity results of MIL-101(Cr) with other catalysts was also performed, and the results are given in Table 3.

**TABLE 3:** The comparison between this catalytic system and previously reported catalytic systems^a^.

Entry	Catalyst	Solvent	Temperature (˚C)	Time (min)	Yield (%)	Ref.
1	NH_2_SO_3_H (50 mol%)	CH_3_CN (5 ml)	61	30	95	Heravi et al. (2010)
2	Nano-SiO_2_ (15 mol%)	CH_3_CN (5 ml)	r.t.	30	96	Mousavi and Maghsoodlou (2015)
3	H_6_P_2_W_18_O_62_—18H_2_O (1 mol%)	CH_3_CN (5 ml)	80	15	96	Heravi et al. (2008)
4^b^	silica gel	-	120	3	95	Krishnamurthy and Jagannath (2013)
5	iodine (10 mol%)	CH_3_CN (5 ml)	reflux	10	81.2	Puligoundla et al. (2013)
6^c^	T@ILs^d^ nanocatalyst (10 mg)	EtOH (5 ml)	reflux	35	92	Bakhshali-Dehkordi et al. (2020)
7^c^	Fe_3_O_4_@TiO_2_-IL (0.008 g)	-	80	45	95	Bakhshali‐Dehkordi et al. (2020)
8	MIL-101(Cr) (0.25 mol%)	acetonitrile (2 ml)	r.t.	30	94	This work

a The data are for performing the reaction using benzaldehyde, 1*H*-1,2,4-triazol-3-amine, and dimedone as substrates.

b Under irradiation with microwaves of 150 W at 120°C and pressure of 100 psi.

c The data are for performing the reaction using *p*-chlorobenzaldehyde, 1*H*-1,2,4-triazol-3-amine, and dimedone.

d T@ILs, nanocatalyst = TiO_2_ nanoparticles supported ionic liquids.

## Conclusion

Finally, we have established a comprehensive and feasible MIL-101(Cr) catalyzed synthesis of structurally diverse quinazolinone heterocycles, which could be exploited for the subsequent preparation of biologically significant compounds. This strategy could produce numerous quinazolinones by employing several mono- or bis-functionalized benzaldehyde compounds in the presence of a highly active catalyst using a low amount of solvent, which also makes our protocol an extremely useful complement to the current procedures for the quinazolinone formation. Moreover, all products were obtained in high yields *via* recrystallization.

## Data Availability

The original contributions presented in the study are included in the article/Supplementary Material; further inquiries can be directed to the corresponding authors.

## References

[B1] Aboonajmi J. Sharghi H. Aberi M. Shiri P. (2020). Consecutive Oxidation/Condensation/Cyclization/Aromatization Sequences Catalyzed by Nanostructured Iron(III)‐Porphyrin Complex towards Benzoxazole Derivatives. Eur. J. Org. Chem. 2020 (37), 5978–5984. 10.1002/ejoc.202000999

[B2] Al-Soud Y. A. Al-Masoudi N. A. Ferwanah A. E.-R. S. (2003). Synthesis and Properties of New Substituted 1,2,4-triazoles: Potential Antitumor Agents. Bioorg. Med. Chem. 11 (8), 1701–1708. 10.1016/s0968-0896(03)00043-9 12659756

[B3] Alizadeh A. Roosta A. Halvagar M. (2019). Four-Component Regio- and Diastereoselective Synthesis of Pyrrolizidines Incorporating Spiro-Oxindole/Indanedione via 1,3-Dipolar Cycloaddition Reaction of Azomethine Ylides. ChemistrySelect 4 (1), 71–74. 10.1002/slct.201803418

[B4] Alizadeh A. Roosta A. Halvagar M. R. (2017). An Efficient One-Pot Synthesis of Highly Substituted [1, 8] Naphthyridin-1-Phenyl-1-Ethanone Derivatives via a Four-Component Reaction. J. Iran. Chem. Soc. 14, 1–9. 10.1007/s13738-017-1152-7

[B5] Alizadeh A. Roosta A. Rezaiyehrad R. Halvagar M. (2017). Efficient One Pot and Chemoselective Synthesis of Functionalized 3-Bromo-4,5-Dihydroisoxazole Derivatives via 1,3-dipolar Cycloaddition Reactions of Nitrile Oxides. Tetrahedron 73 (48), 6706–6711. 10.1016/j.tet.2017.10.003

[B6] Anderson E. B. Long T. E. (2010). Imidazole- and Imidazolium-Containing Polymers for Biology and Material Science Applications. Polymer 51 (12), 2447–2454. 10.1016/j.polymer.2010.02.006

[B7] Bakhshali‐Dehkordi R. Ghasemzadeh M. A. Safaei‐Ghomi J. (2020). Preparation and Characterization of a Novel DABCO‐based Ionic Liquid Supported on Fe3O4@ TiO2 Nanoparticles and Investigation of its Catalytic Activity in the Synthesis of Quinazolinones. Appl. Organomet. Chem. 34 (9), e5721.

[B8] Bakhshali-Dehkordi R. Ghasemzadeh M. A. Safaei-Ghomi J. (2020). Multicomponent Preparation of Quinazolinone Derivatives in the Presence of TiO2 Nanoparticles Supported Ionic Liquids. Polycycl. Aromat. Compd., 1–18.

[B9] Bedard N. Fistrovich A. Schofield K. Shaw A. Hulme C. (2022). Recent Applications of Multicomponent Reactions toward Heterocyclic Drug Discovery. Multicomponent React. towards Heterocycles Concepts Appl., 339–409. 10.1002/9783527832439.ch9

[B10] Biesen L. Müller T. J. J. (2021). Multicomponent and One‐pot Syntheses of Quinoxalines. Adv. Synth. Catal. 363 (4), 980–1006. 10.1002/adsc.202001219

[B11] Dixit P. P. Nair P. S. Patil V. J. Jain S. Arora S. K. Sinha N. (2005). Synthesis and Antibacterial Activity of Novel (Un)substituted Benzotriazolyl Oxazolidinone Derivatives. Bioorg. Med. Chem. Lett. 15 (12), 3002–3005. 10.1016/j.bmcl.2005.04.045 15908210

[B12] Gajaganti S. Kumari S. Kumar D. Allam B. K. Srivastava V. Singh S. (2018). An Efficient, Green, and Solvent-free Multi-Component Synthesis of Benzimidazolo/Benzothiazolo Quinazolinone Derivatives Using Sc (OTf)3Catalyst under Controlled Microwave Irradiation. J. Heterocycl. Chem. 55 (11), 2578–2584. 10.1002/jhet.3314

[B13] Henary M. Kananda C. Rotolo L. Savino B. Owens E. A. Cravotto G. (2020). Benefits and Applications of Microwave-Assisted Synthesis of Nitrogen Containing Heterocycles in Medicinal Chemistry. RSC Adv. 10 (24), 14170–14197. 10.1039/d0ra01378a 35498463 PMC9051880

[B14] Heravi M. M. Ranjbar L. Derikvand F. Alimadadi B. Oskooie H. A. Bamoharram F. F. (2008). A Three Component One-Pot Procedure for the Synthesis of [1,2,4]triazolo/benzimidazolo-Quinazolinone Derivatives in the Presence of H6P2W18O(62).18H2O as a Green and Reusable Catalyst. Mol. Divers 12 (3), 181–185. 10.1007/s11030-008-9086-8 18780153

[B15] Heravi M. M. Derikvand F. Ranjbar L. (2010). Sulfamic Acid-Catalyzed, Three-Component, One-Pot Synthesis of [1,2,4]Triazolo/Benzimidazolo Quinazolinone Derivatives. Synth. Commun. 40 (5), 677–685. 10.1080/00397910903009489

[B16] Karan R. Agarwal P. Sinha M. Mahato N. (2021). Recent Advances on Quinazoline Derivatives: A Potential Bioactive Scaffold in Medicinal Chemistry. ChemEngineering 5 (4), 73. 10.3390/chemengineering5040073

[B17] Keri R. S. Patil M. Budagumpi S. Sasidhar B. S. (2021). An Efficient, Multicomponent Synthesis of Aminoalkylnaphthols via Betti Reaction Using ZSM‐5 as a Recoverable and Reusable Catalyst. Appl. Organomet. Chem. 35 (9), e6316. 10.1002/aoc.6316

[B18] Khangah P. F. HaeriZadeh N. Sirouspour M. (2021). Synthesis of Isoquinoline Derivatives Using Multicomponent Reaction of Isocyanides.

[B19] Koolivand M. Nikoorazm M. Ghorbani‐Choghamarani A. Tahmasbi B. (2021). Cu–citric Acid Metal–Organic Framework: Synthesis, Characterization and Catalytic Application in Suzuki–Miyaura Cross‐coupling Reaction and Oxidation of Sulfides. Appl. Organomet. Chem. 35 (12), e6434. 10.1002/aoc.6434

[B20] Krishnamurthy G. Jagannath K. V. (2013). Microwave-assisted Silica-Promoted Solvent-free Synthesis of Triazoloquinazolinone and Benzimidazoquinazolinones. J. Chem. Sci. 125 (4), 807–811. 10.1007/s12039-013-0398-6

[B21] Kundu N. G. Mahanty J. S. Chowdhury C. Dasgupta S. K. Das B. Spears C. P. (1999). 5-(Acylethynyl)uracils, 5-(Acylethynyl)-2'-Deoxyuridines and 5-(Acylethynyl)-1-(2-Hydroxyethoxy)methyluracils. Their Synthesis, Antiviral and Cytotoxic activities11Part 25 of Our Series of Studies on Uracil Derivatives and Analogues. For Part 24, See [1]. Eur. J. Med. Chem. 34 (5), 389–398. 10.1016/s0223-5234(99)80088-9

[B22] Lipson V. V. Desenko S. M. Shirobokova M. G. Borodina V. V. (2003). Synthesis of 9-Aryl-6,6-Dimethyl-5,6,7,9-Tetrahydro-1,2,4-Triazolo[5,1-B]quinazolin-8(4H)ones. Chem. Heterocycl. Compd. 39 (9), 1213–1217. 10.1023/b:cohc.0000008269.69460.ac

[B23] Liu H. Kanjilal P. Thayumanavan S. (2022). Self‐assembly of Polymers from Multicomponent Reactions. Polym. Int. 71, 562–568. 10.1002/pi.6352

[B24] Ma X. Liu F. Helian Y. Li C. Wu Z. Li H. (2021). Current Application of MOFs Based Heterogeneous Catalysts in Catalyzing Transesterification/esterification for Biodiesel Production: A Review. Energy Convers. Manag. 229, 113760. 10.1016/j.enconman.2020.113760

[B25] Mashayekh K. Shiri P. (2019). An Overview of Recent Advances in the Applications of Click Chemistry in the Synthesis of Bioconjugates with Anticancer Activities. ChemistrySelect 4 (46), 13459–13478. 10.1002/slct.201902362

[B26] Mousavi M. R. Maghsoodlou M. T. (2015). Nano-SiO2: a Green, Efficient, and Reusable Heterogeneous Catalyst for the Synthesis of Quinazolinone Derivatives. J. Iran. Chem. Soc. 12 (5), 743–749. 10.1007/s13738-014-0533-4

[B27] Niknam E. Panahi F. Daneshgar F. Bahrami F. Khalafi-Nezhad A. (2018). Metal-Organic Framework MIL-101(Cr) as an Efficient Heterogeneous Catalyst for Clean Synthesis of Benzoazoles. ACS omega 3 (12), 17135–17144. 10.1021/acsomega.8b02309 31458334 PMC6643801

[B28] Novak I. Abu-Izneid T. Kovač B. Klasinc L. (2009). Electronic Structure and Stability of Benzotriazoles. J. Phys. Chem. A 113 (35), 9751–9756. 10.1021/jp905640b 19708693

[B29] Perregaard J. Arnt J. Boegesoe K. P. Hyttel J. Sanchez C. (1992). Noncataleptogenic, Centrally Acting Dopamine D-2 and Serotonin 5-HT2 Antagonists within a Series of 3-substituted 1-(4-Fluorophenyl)-1h-Indoles. J. Med. Chem. 35 (6), 1092–1101. 10.1021/jm00084a014 1348090

[B30] Puligoundla R. G. Karnakanti S. Bantu R. Nagaiah K. Kondra S. B. Nagarapu L. (2013). A Simple, Convenient One-Pot Synthesis of [1,2,4]triazolo/benzimidazolo Quinazolinone Derivatives by Using Molecular Iodine. Tetrahedron Lett. 54 (20), 2480–2483. 10.1016/j.tetlet.2013.02.099

[B31] Rezaei Z. Khabnadideh S. Pakshir K. Hossaini Z. Amiri F. Assadpour E. (2009). Design, Synthesis, and Antifungal Activity of Triazole and Benzotriazole Derivatives. Eur. J. Med. Chem. 44 (7), 3064–3067. 10.1016/j.ejmech.2008.07.012 18760508

[B32] Sano H. Noguchi T. Miyajima A. Hashimoto Y. Miyachi H. (2006). Anti-angiogenic Activity of Basic-type, Selective Cyclooxygenase (COX)-1 Inhibitors. Bioorg. Med. Chem. Lett. 16 (11), 3068–3072. 10.1016/j.bmcl.2006.02.021 16513348

[B33] Sharghi H. Aboonajmi J. Aberi M. Shiri P. (2018). Heterogeneous AlPO 4 (SO 3 H) Nanosheets: Novel Catalyst for the Multi-Component Synthesis of Quinazolinones and Highly Functionalized Piperidines. J. Iran. Chem. Soc. 15, 1–12. 10.1007/s13738-018-1308-0

[B34] Shiri P. Aboonajmi J. (2020). A Systematic Review on Silica-, Carbon-, and Magnetic Materials-Supported Copper Species as Efficient Heterogeneous Nanocatalysts in "click" Reactions. Beilstein J. Org. Chem. 16 (1), 551–586. 10.3762/bjoc.16.52 32280385 PMC7136568

[B35] Shiri P. Amani A. M. (2021). A Brief Overview of Catalytic Applications of Dendrimers Containing 1,4-Disubstituted-1,2,3-Triazoles. Monatsh Chem. 152 (4), 367–385. 10.1007/s00706-021-02753-3

[B36] Shiri P. Amani A. M. Aboonajmi J. (2021). Supported Cu (II)-Schiff Base: Novel Heterogeneous Catalyst with Extremely High Activity for Eco-Friendly, One-Pot and Multi-Component C–S Bond-Forming Reaction toward a Wide Range of Thioethers as Biologically Active Cores. Mol. Divers., 1–10. 10.1007/s11030-021-10227-1 33978897

[B37] Shiri P. (2020). An Overview on the Copper‐promoted Synthesis of Five‐membered Heterocyclic Systems. Appl. Organomet. Chem. 34 (5), e5600. 10.1002/aoc.5600

[B38] Shiri P. (2021). Novel Hybrid Molecules Based on Triazole-β-Lactam as Potential Biological Agents. Mrmc 21 (5), 536–553. 10.2174/1389557520666201027160436 33109046

[B39] Von Angerer E. Strohmeier J. 2-Phenylindoles (1987). 2-Phenylindoles. Effect of N-Benzylation on Estrogen Receptor Affinity, Estrogenic Properties, and Mammary Tumor Inhibiting Activity. J. Med. Chem. 30 (1), 131–136. 10.1021/jm00384a022 3806590

[B40] Wang R. Twamley B. Shreeve J. n. M. (2006). A Highly Efficient, Recyclable Catalyst for C−C Coupling Reactions in Ionic Liquids: Pyrazolyl-Functionalized N-Heterocyclic Carbene Complex of Palladium(II). J. Org. Chem. 71 (1), 426–429. 10.1021/jo052098b 16388677

[B41] Wang Z. Liang M. Hao Y. Zhang Y. Wang L. Sun Z. (2013). Influence of the N-Heterocycle Substituent of the Dithieno[3,2-B:2′,3′-D]pyrrole (DTP) Spacer as Well as Sensitizer Adsorption Time on the Photovoltaic Properties of Arylamine Organic Dyes. J. Mat. Chem. A 1 (38), 11809–11819. 10.1039/c3ta12746j

[B42] Wiglenda T. Gust R. (2007). Structure−Activity Relationship Study to Understand the Estrogen Receptor-dependent Gene Activation of Aryl- and Alkyl-Substituted 1H-Imidazoles. J. Med. Chem. 50 (7), 1475–1484. 10.1021/jm061106t 17352461

[B43] Wiglenda T. Ott I. Kircher B. Schumacher P. Schuster D. Langer T. (2005). Synthesis and Pharmacological Evaluation of 1H-Imidazoles as Ligands for the Estrogen Receptor and Cytotoxic Inhibitors of the Cyclooxygenase. J. Med. Chem. 48 (20), 6516–6521. 10.1021/jm050190u 16190777

[B44] Yadav D. K. T. Rajak S. S. Bhanage B. M. (2014). N-arylation of Indoles with Aryl Halides Using Copper/glycerol as a Mild and Highly Efficient Recyclable Catalytic System. Tetrahedron Lett. 55 (4), 931–935. 10.1016/j.tetlet.2013.12.053

