# Supplementary material for: A Quick Access to Structurally Diverse Triazoloquinazoline Heterocycles via the MIL-101(Cr)-Catalyzed One-Pot Multi-Component Reaction of a Series of Benzaldehydes, Dimedone, and 1H-1,2,4-Triazol-3-Amine Under Green Conditions
Source: Front Chem. 2022 Jul 25;10:898658. doi: 10.3389/fchem.2022.898658 (PMC9357931; doi:10.3389/fchem.2022.898658)
Supplement: Supplementary file 1 [file Table1.DOCX]

Supplementary Material

# Supplementary Data

**Experimental Section**

**Instrumentation, analyses and starting materials**

Chemical materials and solvents were either synthesized in our laboratory or purchased from Fluka, Aldrich and Merck Companies. NMR spectra were recorded on a Bruker Avance DPX-250 (^1^H-NMR 250 and 400 MHz and ^13^C-NMR 62.9 and 100 MHz) spectrometer in pure deuterated solvents with tetramethylsilane as an internal standard. The purity determination of the starting materials as well as monitoring of reactions were accomplished by TLC on silica gel PolyGram SILG/UV 254 plates. Column Chromatography was performed on short columns of silica gel 60 (70-230 mesh) in glass columns.

**Synthetic route for MIL-101(Cr) catalyst**

MIL-101(Cr) catalyst was synthesized according to our previously reported procedure. First, a mixture of Cr(NO_3_)_3_·9H_2_O (5.4 g) and terephthalic acid (1.5 g) was added to deionized water (45 mL) and hydrofluoric acid (0.6 mL, 5 mol L^−1^) in a Teflon-lined stainless steel autoclave. After sonification for 10 min, the mixture was heated in an oven at 220 ˚C for 9 h. In continuation, the mixture was cool down to r.t. and then the mixture was filtered and washed several times with hot water and hot DMF. The resulting MIL-101(Cr) catalyst was dried and purified more with hot filtration in DMF at 120 ˚C for 12 h. The solid was washed several times with hot DMF and hot ethanol. Finally, the solid was filtered and dried at 80 ˚C for 6 h. MIL-101(Cr) catalyst was characterized as mentioned in the results and discussion.

**General procedure for the one-pot multi‑component synthesis of structurally diverse quinazolinone heterocycles**

MIL-101(Cr) as catalyst was added to a mixture of aldehyde (1.0 mmol), dimedone (1.0 mmol), 1*H*-1,2,4-triazol-3-amine (1.0 mmol) in acetonitrile (2.0 mL) and stirred at r.t. for an appropriate time. After the completion of the reaction, catalyst was separated by centrifugation which was used for the next run. After evaporation of the solvent, product was obtained using recrystallization of the solid residue in hot ethanol.

**6,6-Dimethyl-9-phenyl-5,6,7,9-tetrahydro-[1,2,4]triazolo[5,1 *b*]quinazolin-8(4*H*)-one (4a):** White solid; 94% yield; mp 247–250 °C. ^1^H NMR (250 MHz, CDCl_3_) δ (ppm): 11.76 (s, 1H, NH); 7.61 (s, 1H, =CH), 7.16-7.26 (m, 5H, ArH), 6.38 (s, 1H, CH), 2.51 (s, 2H, CH_2_), 2.22 (s, 2H, CH_2_), 1.09 (s, 3H, CH_3_), 1.03 (s, 3H, CH_3_). ^13^C NMR (62.9 MHz, CDCl_3_) δ (ppm): 193.8, 148.9, 148.4, 147.3, 140.5, 128.6, 128.3, 127.1, 107.4, 58.8, 50.3, 40.6, 32.7, 29.0, 27.6. FT-IR (KBr cm^-1^): 732, 1257, 1365, 1573, 1651, 2965, 3085, 3224.

**6,6-Dimethyl-9-(*p*-tolyl)-5,6,7,9-tetrahydro-[1,2,4]triazolo[5,1 *b*]quinazolin-8(4*H*)-one (4b):** Pale yellow solid; 91% yield; mp 264–266 C; ^1^H NMR (250 MHz, DMSO-*d_6_*) δ (ppm): 11.08 (s, 1H, NH). 7.66 (s, 1H, =CH), 7.06 (s, 4H, ArH), 6.15 (s, 1H, CH), 2.49 (s, 2H, CH_2_), 2.24-2.02 (m, 5H, CH_2_ and CH_3_), 1.03 (s, 3H, CH_3_), 0.95 (s, 3H, CH_3_). ^13^C NMR (62.9 MHz, DMSO-*d_6_*) δ (ppm): 192.8, 150.1, 149.8, 146.7, 138.7, 136.8, 128.7, 126.7, 105.6, 57.5, 49.7, 32.1, 28.4, 26.7, 20.5. FT-IR (KBr cm^-1^): 735, 1249, 1365, 1581, 1651, 2923, 3085, 3240.

**9-(4-Methoxyphenyl)-6,6-dimethyl-5,6,7,9-tetrahydro-[1,2,4]triazolo[5,1-*b*]quinazolin-8(4*H*)-one (4c):** Pale yellow solid; 89% yield; mp 230–232 °C; ^1^H NMR (250 MHz, DMSO-*d_6_*) δ (ppm): 11.08 (s, 1H, NH), 7.66 (s, 1H, =CH), 7.10 (d, 2H, *J*=10.0 Hz, ArH), 6.81 (d, 2H, *J*=10.0 Hz, ArH), 6.14 (s, 1H, CH), 3.68 (s, 3H, OCH_3_), 2.53-2.48 (m, 2H, CH_2_), 2.21 (d, 1H, *J*=15.0 Hz), 2.06 (d, 1H, *J*=15.0 Hz), 1.03 (s, 3H, CH_3_), 0.96 (s, 3H, CH_3_). ^13^C NMR (62.9 MHz, DMSO-*d_6_*) δ (ppm): 192.9, 158.6, 150.1, 149.9, 146.7, 133.8, 128.0, 113.5, 105.7, 57.3, 54.9, 49.7, 32.1, 28.5, 26.8. FT-IR (KBr cm^-1^): 1249, 1365, 1573, 1651, 2923, 3085.

**9-(4-Fluorophenyl)-6,6-dimethyl-5,6,7,9-tetrahydro-[1,2,4]triazolo[5,1-*b*]quinazolin-8(4*H*)-one (4d):** Pale yellow solid; 90% yield; mp 257–259 °C; ^1^H NMR (250 MHz, DMSO-*d_6_*) δ (ppm): 11.18 (s, 1H, NH), 7.71 (s, 1H, =CH), 7.26-7.22 (m, 2H, ArH), 7.14-7.09 (m, 2H, ArH), 6.24 (s, 1H, CH), 2.55 (d, 2H, *J*=8.0 Hz, CH_2_), 2.16 (q, 2H, *J*=16.0 Hz, CH_2_), 1.05 (s, 3H, CH_3_), 0.97 (s, 3H, CH_3_). ^13^C NMR (62.9 MHz, DMSO-*d_6_*) δ (ppm): 192.9. 162.6, 160.2, 150.2 (*J*_C-F_= 33.0 Hz), 146.7, 137.8 (*J*_C-F_= 3.0 Hz), 128.9 (*J*_C-F_= 8.0 Hz), 115.0 (*J*_C-F_= 21.0 Hz), 105.3, 57.2, 49.7, 32.1, 28.3, 26.8. FT-IR (KBr cm^-1^): 763, 1218, 1365, 1581, 1651, 2954, 3132.

**9-(4-Chlorophenyl)-6,6-dimethyl-5,6,7,9-tetrahydro-[1,2,4]triazolo[5,1-*b*]quinazolin-8(4*H*)-one (4e):** Pale yellow solid; 92% yield; mp 305–306 °C; ^1^H NMR (250 MHz, DMSO-*d_6_*) δ (ppm): 10.91 (s, 1H, NH); 7.44 (s, 1H, =CH), 7.08 (d, 2H, *J*=7.5 Hz, ArH), 6.95 (d, 2H, *J*=7.5 Hz, ArH), 5.97 (s, 1H, CH), 2.26 (d, 2H, *J*=10.0 Hz, CH_2_), 1.89 (q, 2H, *J*=17.5 Hz, CH_2_), 0.78 (s, 3H, CH_3_), 0.70 (s, 3H, CH_3_). ^13^C NMR (62.9 MHz, DMSO-*d_6_*) δ (ppm): 192.9. 150.5, 150.1, 146.7, 140.4, 132.2, 128.8, 128.2, 105.1, 99.5, 57.3, 49.7, 32.1, 28.3, 26.8. FT-IR (KBr cm^-1^): 763; 1257, 1365, 1573, 1651, 2970, 3085.

**6,6-Dimethyl-9-(4-nitrophenyl)-5,6,7,9-tetrahydro-[1,2,4]triazolo[5,1-*b*]quinazolin-8(4*H*)-one (4f):** yellow solid; 95% yield; 289-291 °C; ^1^H NMR (250 MHz, DMSO-*d_6_*) δ (ppm): 11.30 (s, 1H, NH); 8.14 (d, 2H, *J*=7.5 Hz, ArH), 7.72 (s, 1H, =CH), 7.47 (d, 2H, *J*=7.5 Hz, ArH), 6.35 (s, 1H, CH), 2.51 (d, 2H, *J*=12.5 Hz, CH_2_), 2.13 (q, 2H, *J*=17.5 4 Hz, CH_2_), 1.02 (s, 3H, CH_3_), 0.94 (s, 3H, CH_3_). ^13^C NMR (62.9 MHz, DMSO-*d_6_*) δ (ppm): 192.9, 150.9, 150.4, 148.3, 146.9, 146.8, 128.4, 123.5, 104.7, 57.5, 49.6, 32.2, 28.2, 26.9. FT-IR (KBr cm^-1^): 732, 1249, 1350, 1573, 1643, 2947, 3085.

**6,6-Dimethyl-9-(*o*-tolyl)-5,6,7,9-tetrahydro-[1,2,4]triazolo[5,1-*b*]quinazolin-8(4*H*)-one (4g):** White solid; 88% yield; mp 298–300 °C; ^1^H NMR (250 MHz, DMSO-*d_6_*) δ (ppm): 11.08 (s, 1H, NH); 7.62 (s, 1H, =CH), 7.09–6.95 (m, 4H, ArH), 6.41 (s, 1H, CH), 2.60–2.54 (m, 5H, CH_3_ and CH_2_), 2.11 (q, 2H, *J*=17.5 Hz, CH_2_), 1.02 (s, 3H, CH_3_), 0.96 (s, 3H, CH_3_). ^13^C NMR (62.9 MHz, DMSO-*d_6_*) δ (ppm): 193.0, 150.4, 149.9, 146.5, 140.3, 135.5, 129.9, 127.4, 126.6, 126.2, 106.1, 54.1, 49.7, 32.2, 28.5, 26.8, 18.9. FT-IR (KBr cm^-1^): 748, 1257, 1365, 1581, 1643, 2925, 3085.

**9-(3,4-Dimethoxyphenyl)-6,6-dimethyl-5,6,7,9-tetrahydro-[1,2,4]triazolo[5,1-*b*]quinazolin-8(4*H*)-one (4h):** White solid; 85% yield; mp 218–220 °C; ^1^H NMR (250 MHz, DMSO-*d_6_*) δ (ppm): 11.05 (s, 1H, NH), 7.66 (s, 1H, =CH), 6.84-6.79 (m, 2H, ArH), 6.68-6.63 (m, 1H, ArH), 6.14 (s, 1H, CH), 3.66 (2s, 6H, 2OCH_3_), 2.60-2.49 (m, 2H, CH_2_), 2.21 (d, 1H, *J*=15.0 Hz), 2.05 (d, 1H, *J*=17.5 Hz), 1.02 (s, 3H, CH_3_), 0.97 (s, 3H, CH_3_). ^13^C NMR (62.9 MHz, DMSO-*d_6_*) δ (ppm): 191.9, 170.9, 149.2, 148.8, 147.2, 145.6, 133.0, 118.0, 110.4, 109.8, 104.5, 56.4, 54.3, 48.7, 31.0, 27.5, 25.5, 19.9. FT-IR (KBr cm^-1^): 740, 1257, 1365, 1581, 1651, 2931, 3078.

**9-(4-Hydroxyphenyl)-6,6-dimethyl-5,6,7,9-tetrahydro-[1,2,4]triazolo[5,1-*b*]quinazolin-8(4*H*)-one (4i):** Pale yellow solid; 87% yield; mp 306–307 °C; ^1^H NMR (250 MHz, DMSO-*d_6_*) δ (ppm): 11.04 (s, 1H, NH); 9.40 (s, 1H, OH), 7.66 (s, 1H, =CH), 6.99 (d, 2H, *J*=7.5 Hz, ArH), 6.65 (d, 2H, *J*=10.0 Hz, ArH), 6.11 (s, 1H, CH), 2.55 (d, 1H, *J*=17.5 Hz), 2.47 (d, 1H, *J*=17.5 Hz), 2.12 (q, 2H, *J*=17.5 Hz, CH_2_), 1.03 (s, 3H, CH_3_), 0.96 (s, 3H, CH_3_). ^13^C NMR (62.9 MHz, DMSO-*d_6_*) δ (ppm): 191.9. 155.9, 149.0, 148.8, 145.7, 131.2, 127.1, 113.9, 104.9, 56.4, 48.8, 31.1, 27.5, 25.8. FT-IR (KBr cm^-1^): 732, 1257, 1365, 1573, 1635, 2931, 3085, 3209.

**9-(4-(Dimethylamino)phenyl)-6,6-dimethyl-5,6,7,9-tetrahydro-[1,2,4]triazolo[5,1-*b*]quinazolin-8(4*H*)-one (4j):** Pale yellow solid; 84% yield; mp 286–287 °C; ^1^H NMR (250 MHz, DMSO-*d_6_*) δ (ppm): 10.98 (s, 1H, NH), 7.62 (s, 1H,=CH), 6.96 (d, 2H, *J*=7.5 Hz, ArH), 6.56 (d, 2H, *J*=7.5 Hz, ArH), 6.06 (s, 1H, CH), 2.80 (s, 3H, N–CH_3_), 2.79 (s, 3H, N–CH_3_), 2.54 (d, 2H, *J*= 17.5 Hz, CH_2_), 2.19 (d, 1H, *J*=17.5 Hz), 2.03 (d, 1H, *J*=17.5 Hz), 1.01 (s, 3H, CH_3_), 0.95 (s, 3H, CH_3_). ^13^C NMR (62.9 MHz, DMSO-*d_6_*) δ (ppm): 192.9, 149.8, 149.7, 146.7, 129.3, 127.5, 111.8, 105.9, 57.3, 49.8, 40.0, 32.1, 28.6, 26.7. FT-IR (KBr cm^−1^): 732, 1257, 1365, 1581, 1651, 2923, 3085, 3247.

**9-(2-Chlorophenyl)-6,6-dimethyl-5,6,7,9-tetrahydro-[1,2,4]-triazolo[5,1-*b*]quinazolin-8(4*H*)-one (4k):** Pale yellow solid; 89% yield; mp 287–290 °C; ^1^H NMR (250 MHz, DMSO-*d_6_*) δ (ppm): 11.22 (s, 1H, NH), 7.67 (s, 1H, =CH), 7.38–7.23 (m, 4H, ArH), 6.57 (s, 1H, CH), 2.62–2.53 (m, 2H, CH_2_), 2.21 (d, 1H, *J*=20.0 Hz), 2.07–2.05 (m, 1H), 1.04 (s, 3H, CH_3_), 0.99 (s, 3H, CH_3_). ^13^C NMR (62.9 MHz, DMSO-d6) δ (ppm): 191.8, 150.0, 149.0, 145.8, 137.1, 128.5, 128.3, 126.1, 116.9, 103.4, 55.3, 48.7, 31.0, 27.4, 25.7. FT-IR (KBr cm^-1^): 748, 1257, 1365, 1573, 1643, 2923, 3085, 3224.

**6,6-Dimethyl-9-(naphthalene-1-yl)-5,6,7,9-tetrahydro-[1,2,4]triazolo[5,1-*b*]quinazolin-8(4*H*)-one (4l):** Light yellow solid; 81% yield; mp 300–301 °C; ^1^H NMR (250 MHz, DMSO-*d_6_*) δ (ppm): 11.25 (s, 1H, NH), 8.56 (s, 1H, =CH), 7.93 (d, 1H, *J*=7.5 Hz, ArH), 7.83 (d, 1H, *J*=7.5, ArH), 7.66–7.52 (m, 3H, ArH), 7.47–7.35 (m, 2H, ArH), 7.12 (s, 1H, CH), 2.65 (s, 2H, CH_2_), 2.23 (d, 1H, *J*=17.5 Hz), 2.08 (d, 1H, *J*=15.0 Hz), 1.08 (s, 3H, CH_3_), 1.04 (s, 3H, CH_3_). ^13^C NMR (62.9 MHz, DMSO-*d_6_*) δ (ppm): 193.1, 150.8, 149.8, 146.5, 138.4, 133.3, 130.8, 128.4, 128.3, 126.2, 125.8, 125.4, 123.9, 106.1, 49.9, 32.3, 28.5, 27.1. FT-IR (KBr cm^-1^): 779, 1249, 1365, 1573, 1643, 2923, 3078.

**6,6-Dimethyl-9-(3-nitrophenyl)-5,6,7,9-tetrahydro-[1,2,4]triazolo[5,1-*b*]quinazolin-8(4*H*)-one (4m):** Yellow solid; 90% yield; mp 267–268 °C; ^1^H NMR (250 MHz, DMSO-*d_6_*) δ (ppm): 11.31 (s, 1H, NH), 8.13–8.05 (m, 2H, ArH), 7.73 (s, 1H, =CH), 7.68–7.56 (m, 2H, ArH), 6.41 (s, 1H, CH), 2.57 (s, 2H, CH_2_), 2.22 (d, 1H, *J*=17.5 Hz), 2.08 (d, 1H, *J*=17.5 Hz), 1.04 (s, 3H, CH_3_), 0.97 (s, 3H, CH_3_). ^13^C NMR (62.9 MHz, DMSO-*d_6_*) δ (ppm): 193.0, 151.0, 150.3, 147.5, 146.7, 143.4, 133.6, 130.0, 122.7, 121.6, 104.5, 57.3, 49.6, 32.2, 28.3, 26.8. FT-IR (KBr cm^-1^): 732, 1257, 1350, 1527, 1643, 2930, 3075.


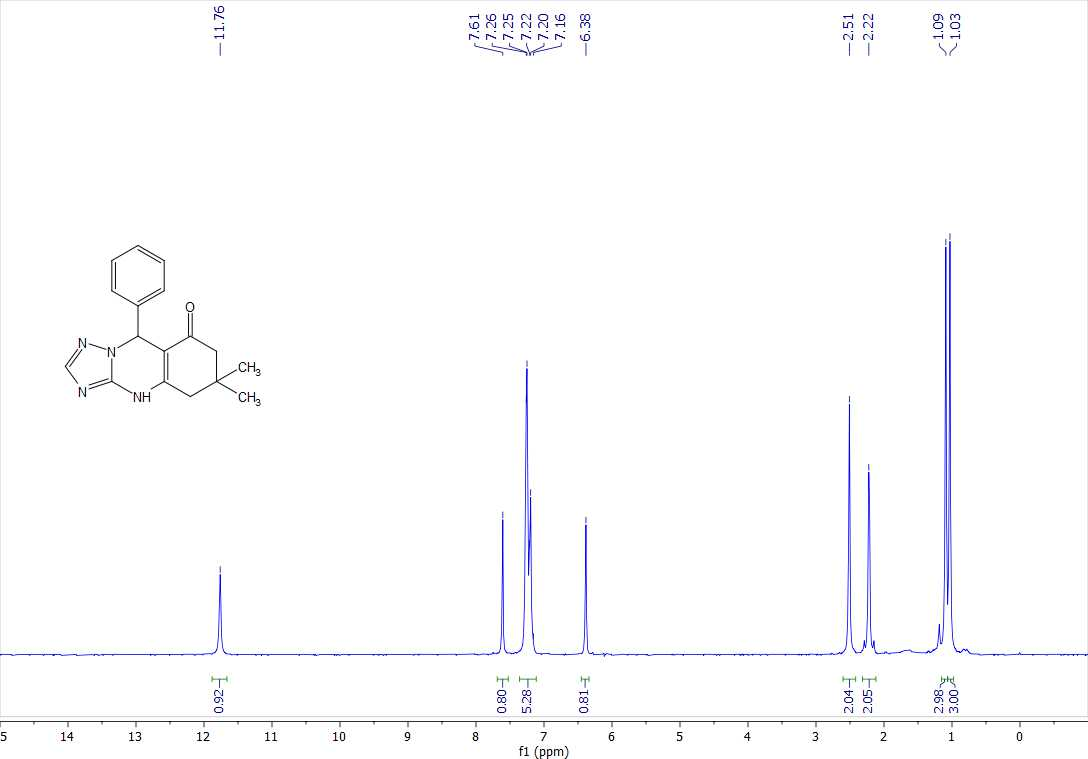


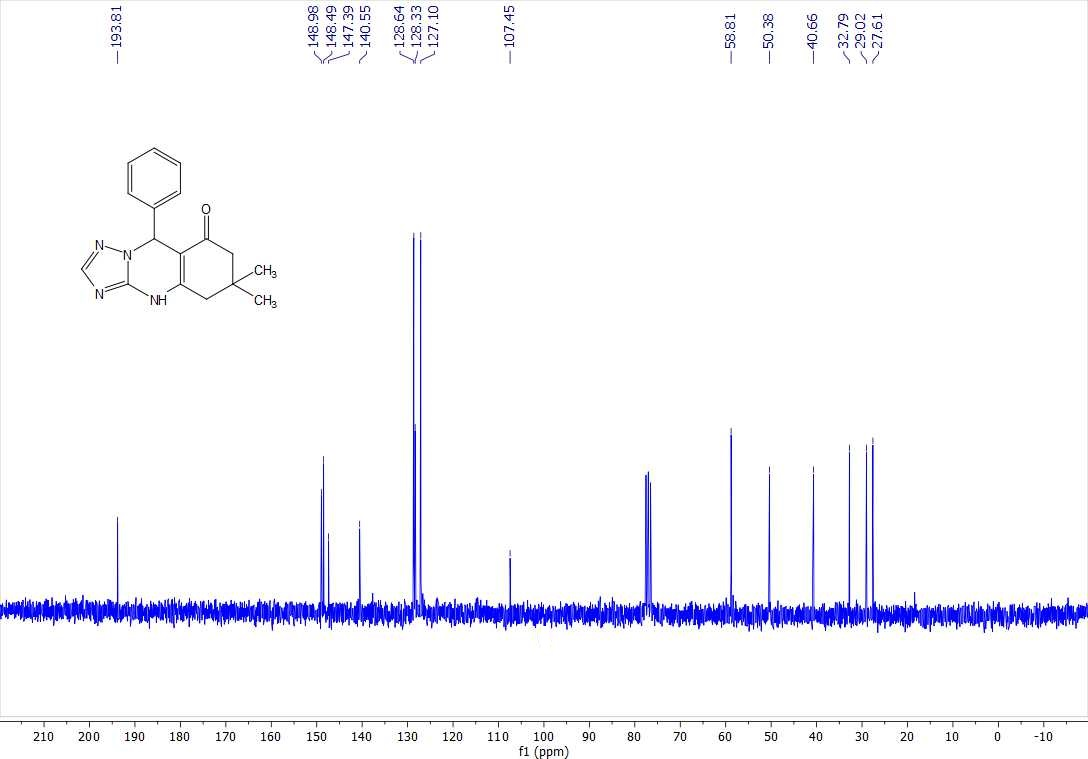


**Scheme 1.** ^1^HNMR and ^13^CNMR for **4a**


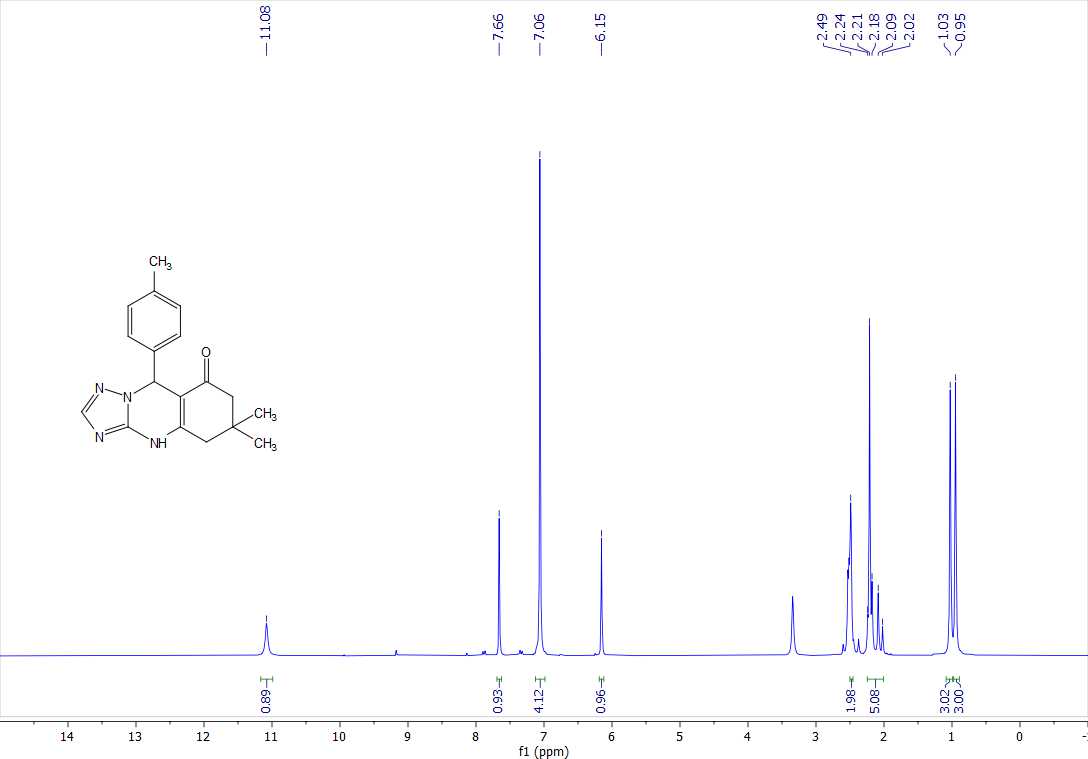


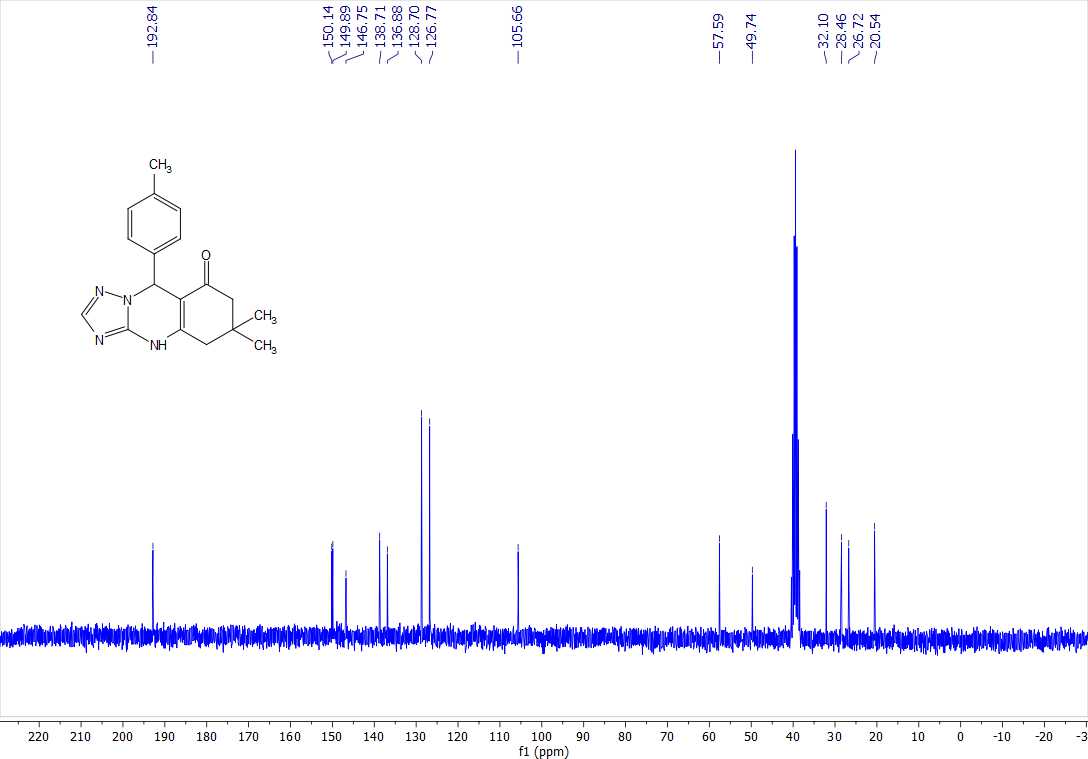


**Scheme 2.** ^1^HNMR and ^13^CNMR for **4b**


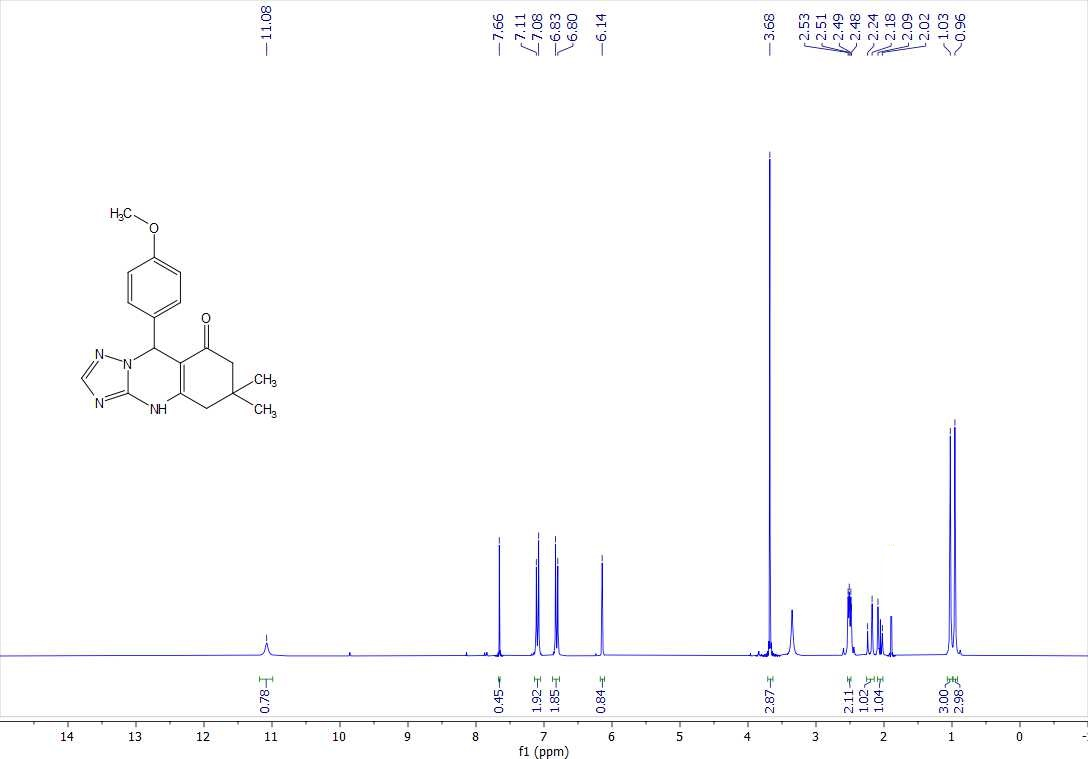


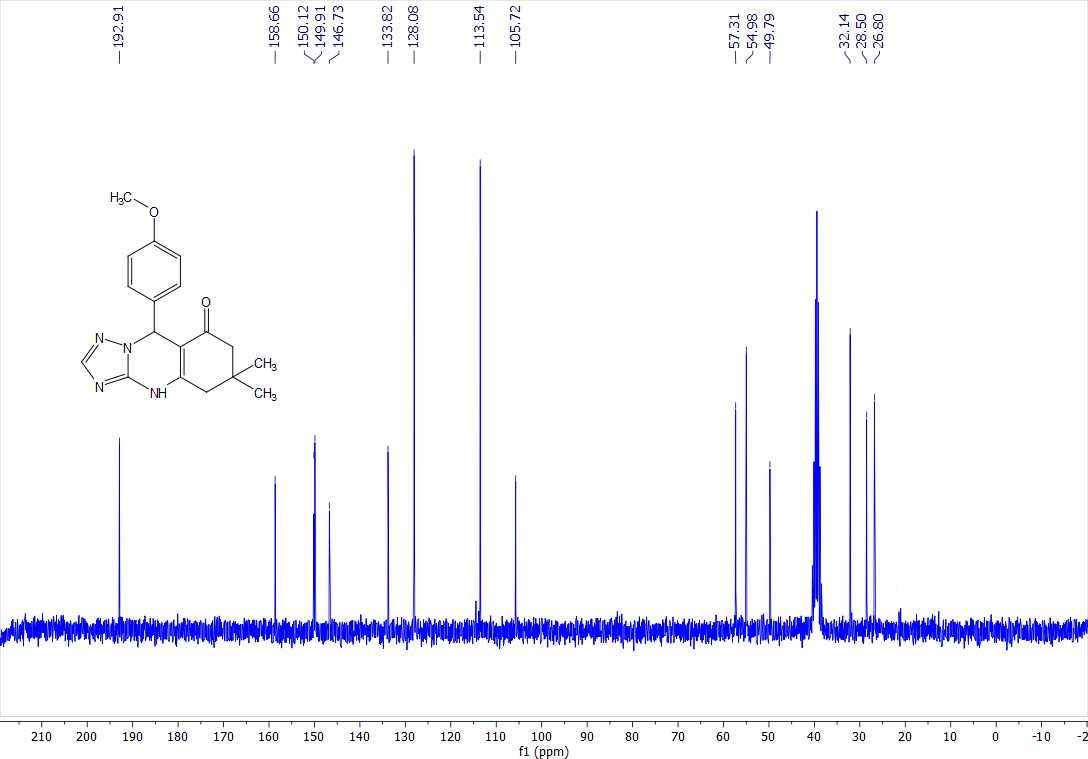


**Scheme 3.** ^1^HNMR and ^13^CNMR for **4c**


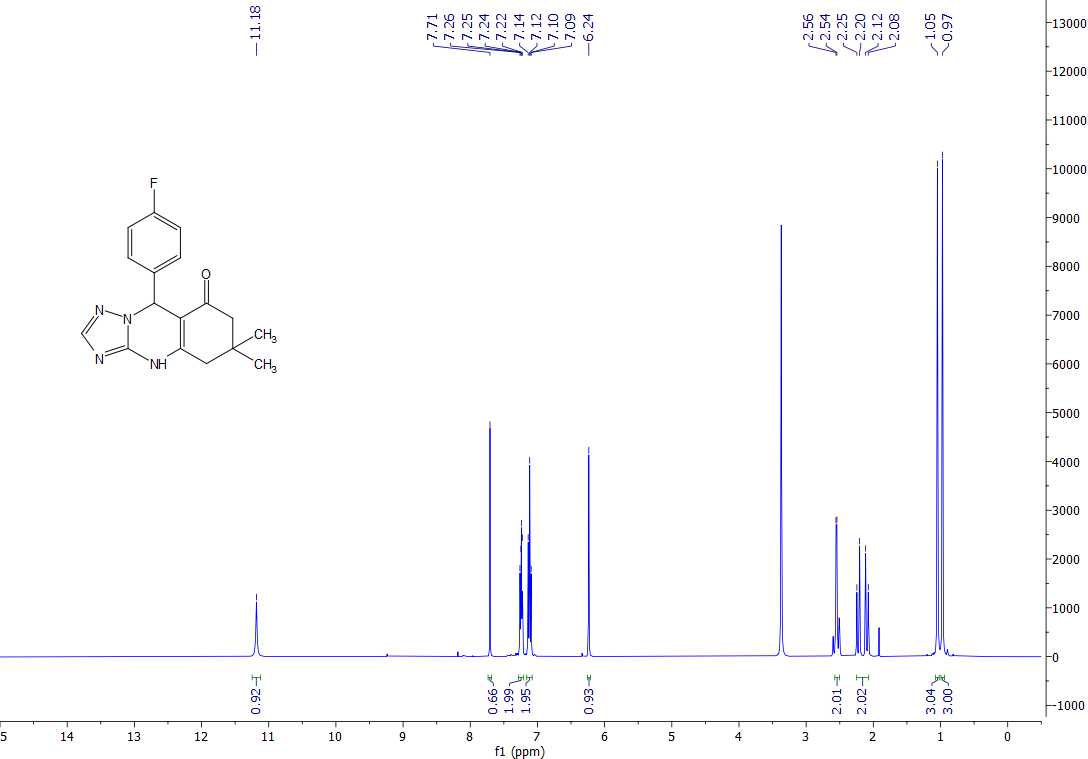


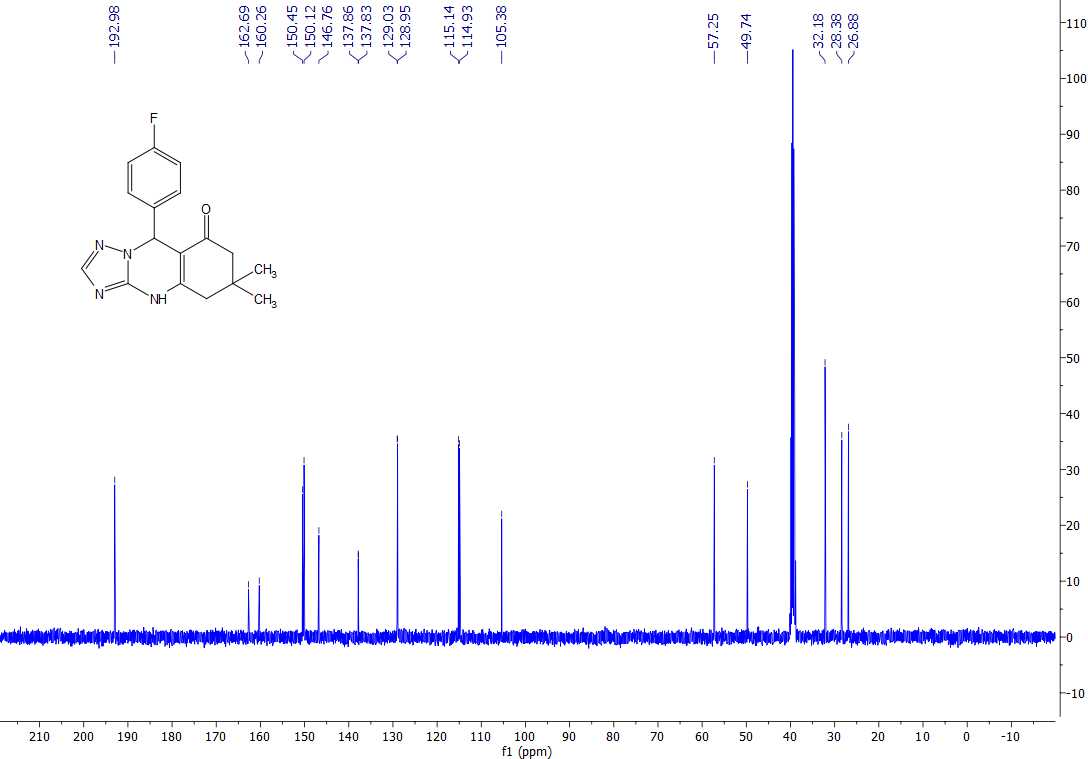


**Scheme 4.** ^1^HNMR and ^13^CNMR for **4d**


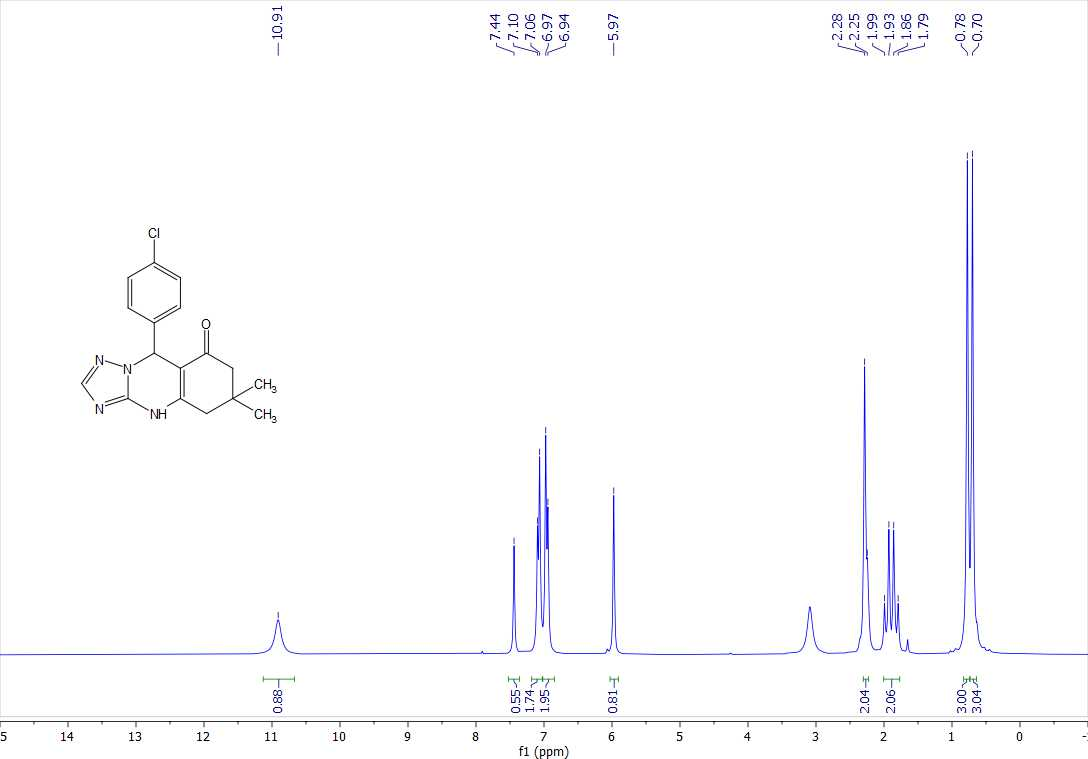


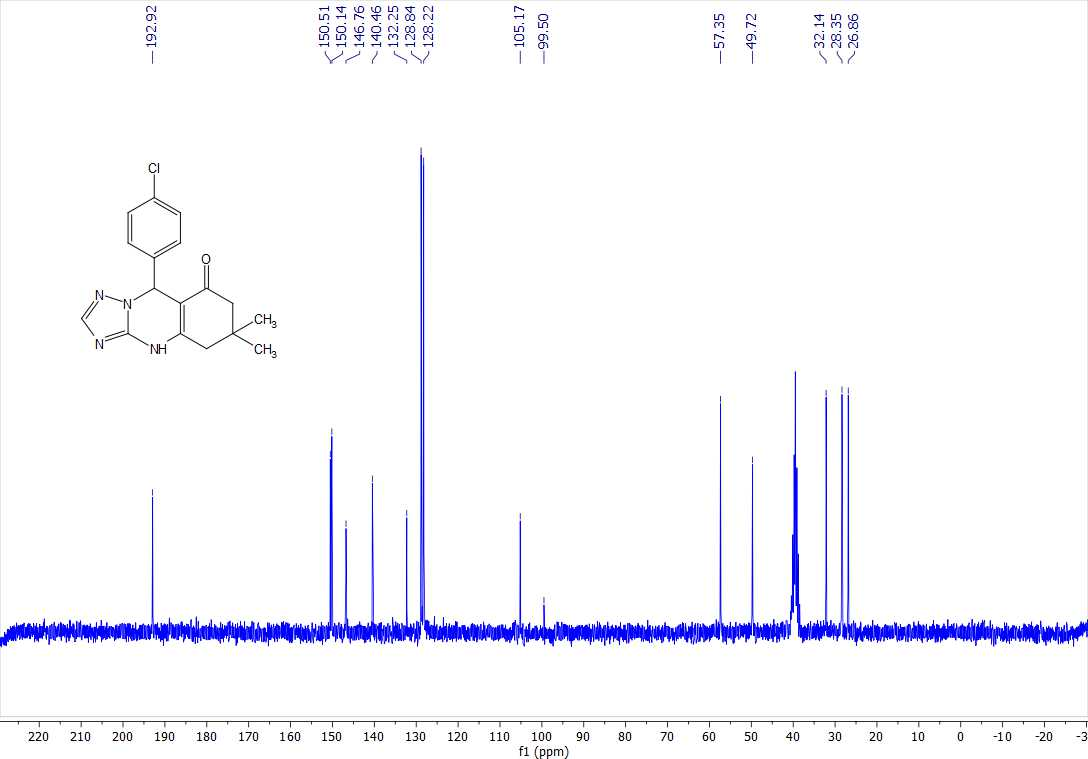


**Scheme 5.** ^1^HNMR and ^13^CNMR for **4e**


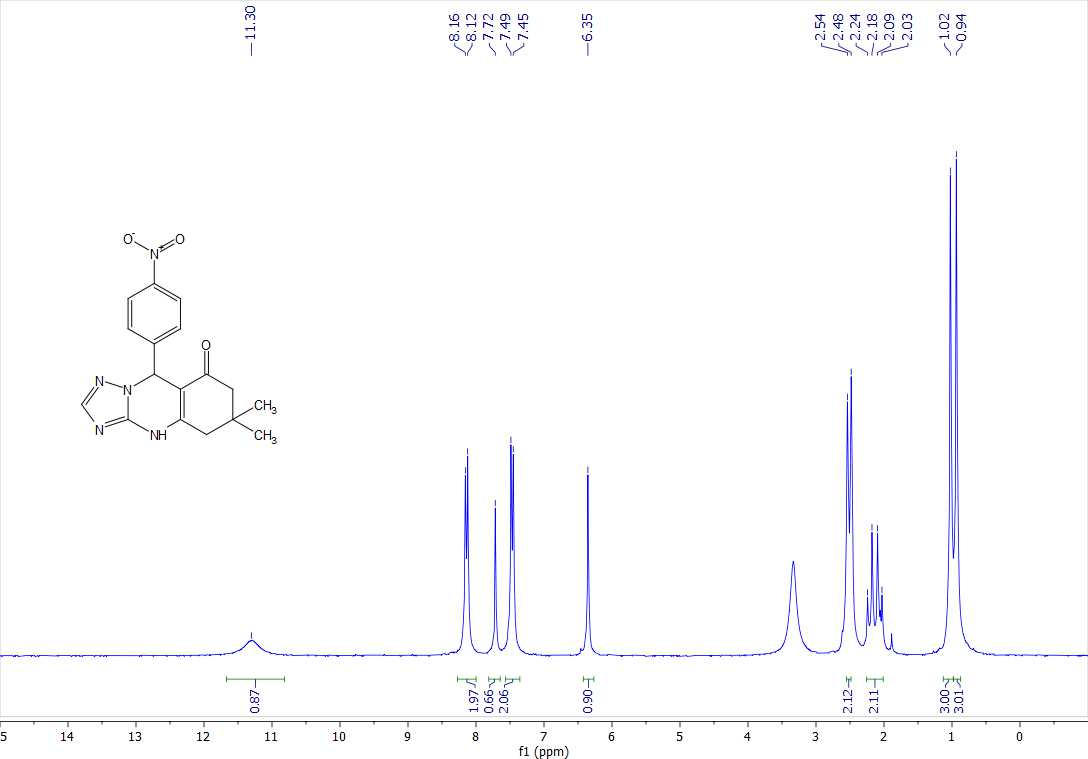


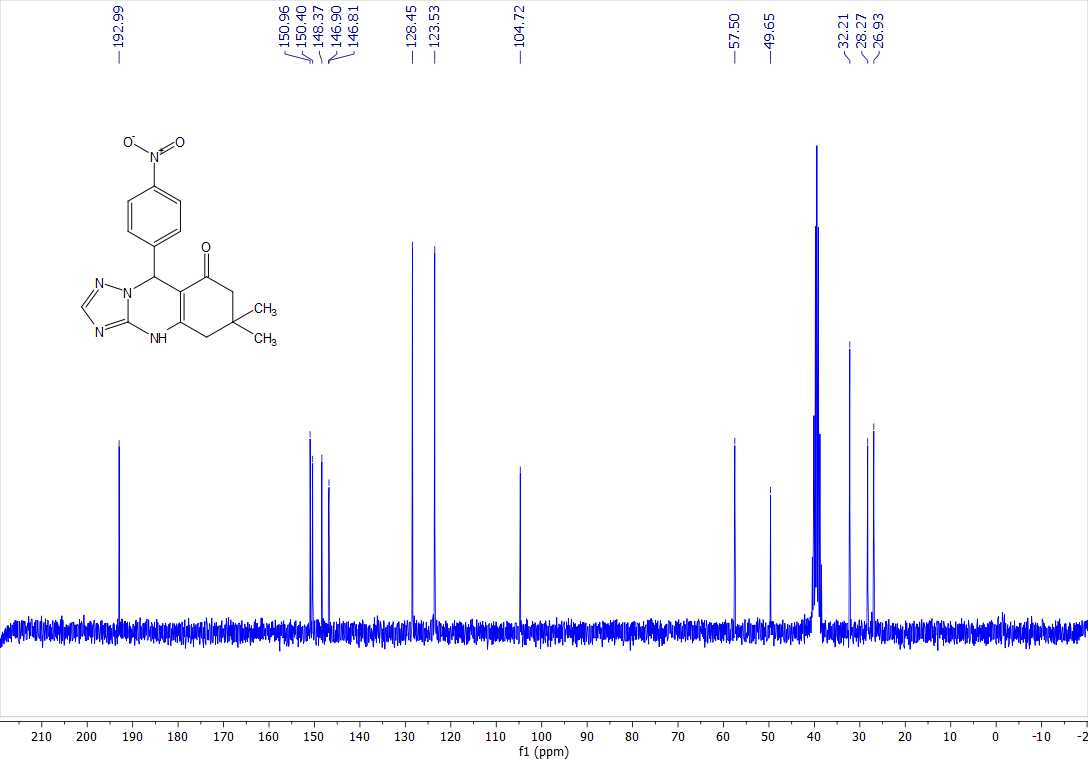


**Scheme 6.** ^1^HNMR and ^13^CNMR for **4f**


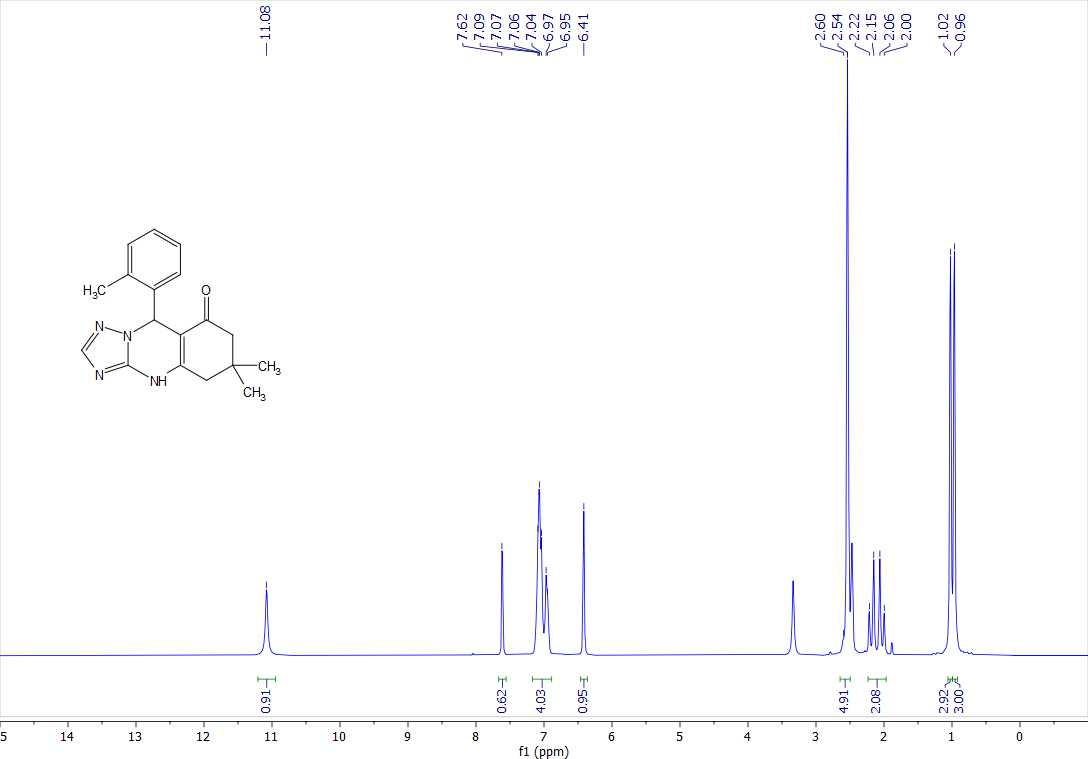


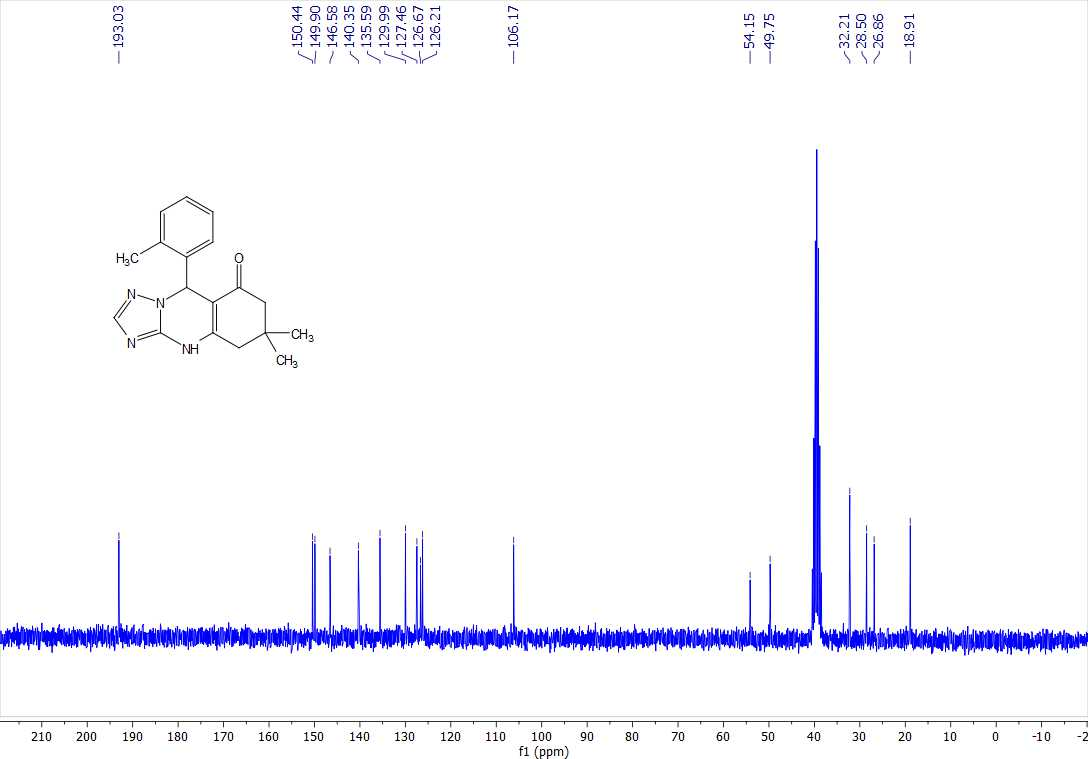


**Scheme 7.** ^1^HNMR and ^13^CNMR for **4g**


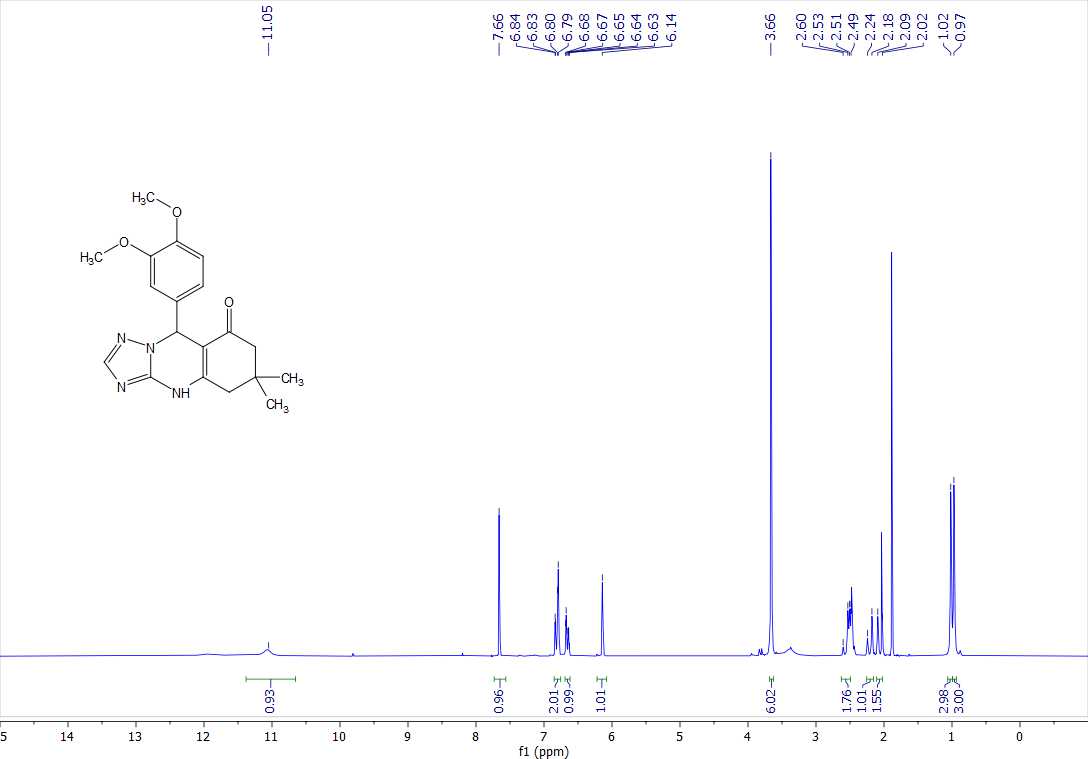


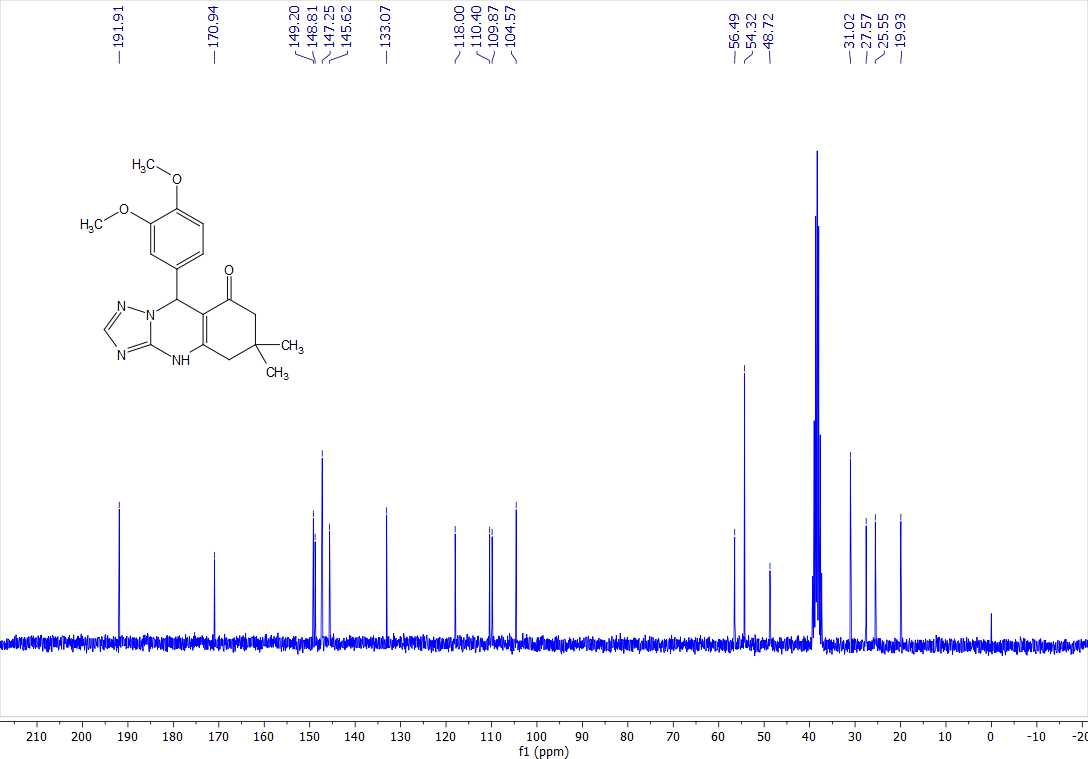


**Scheme 8.** ^1^HNMR and ^13^CNMR for **4h**


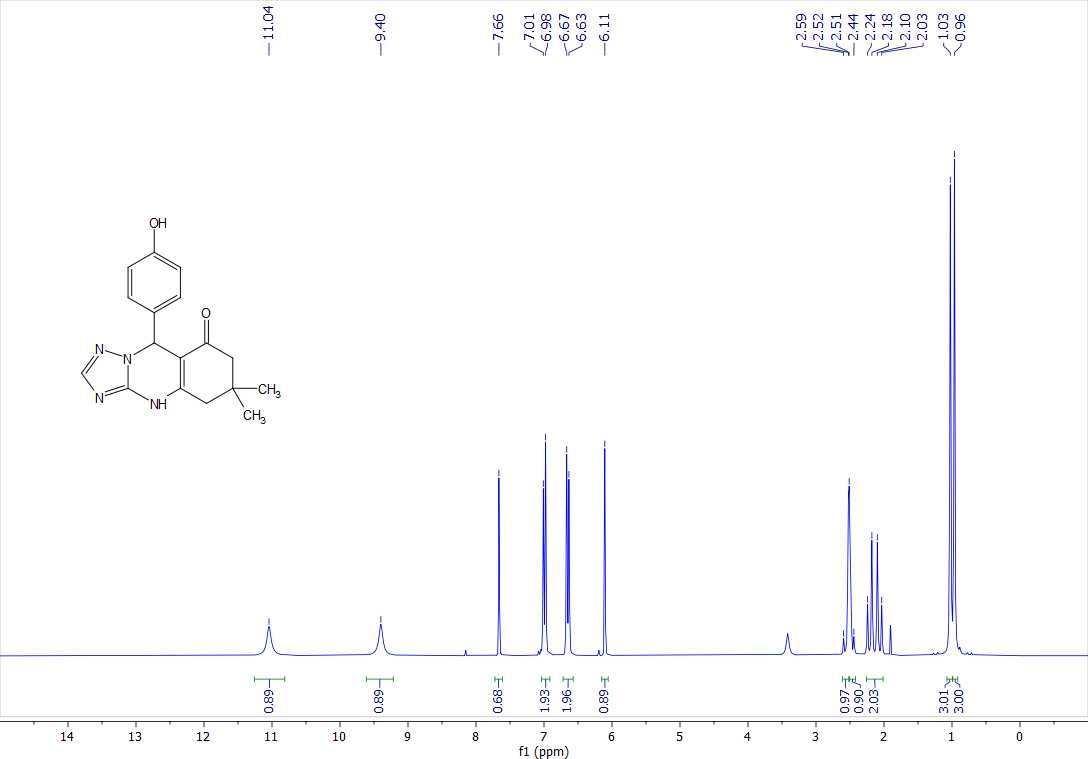


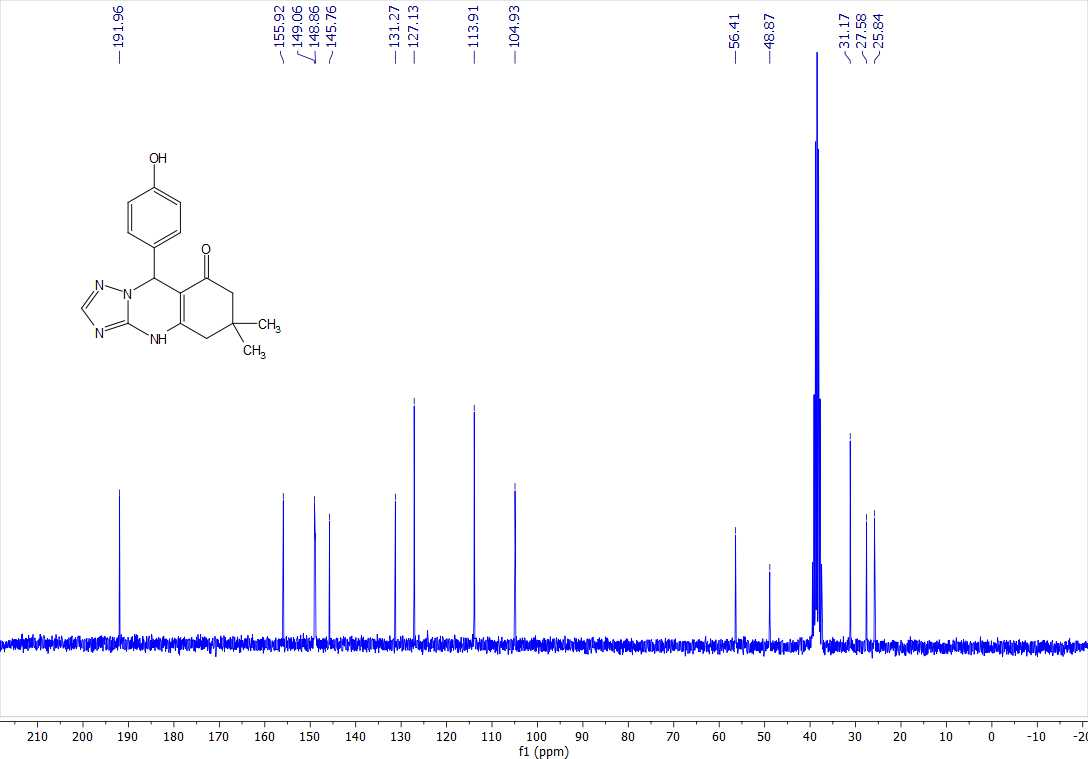


**Scheme 9.** ^1^HNMR and ^13^CNMR for **4i**


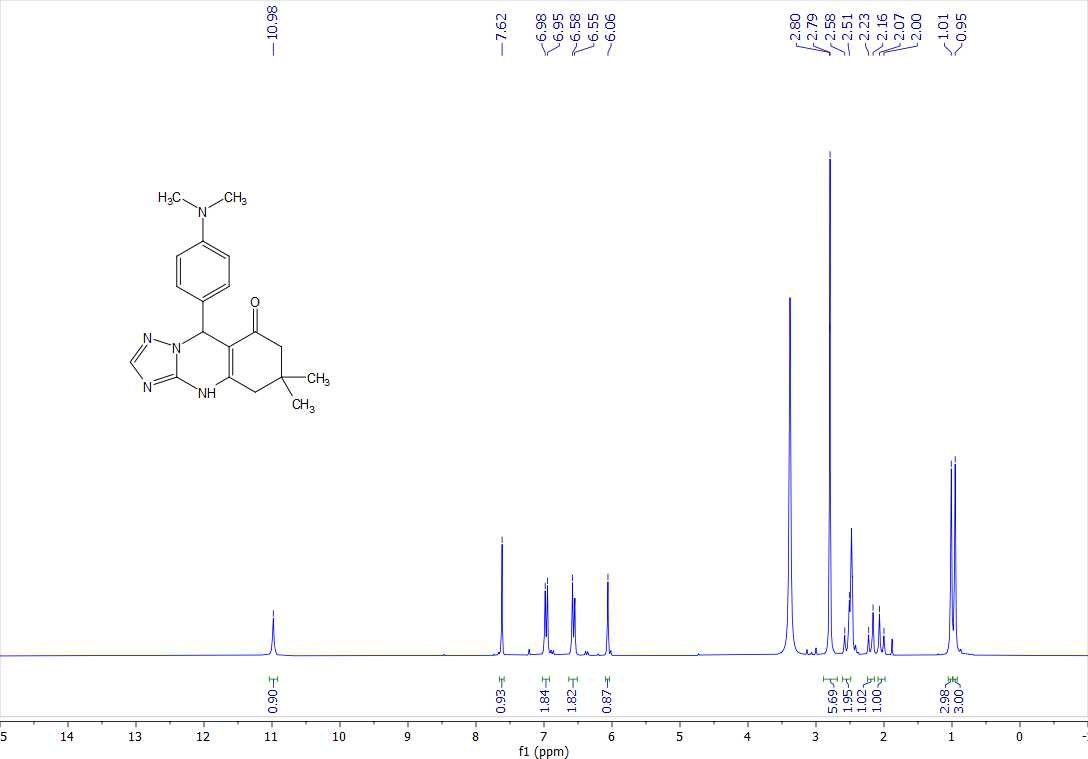


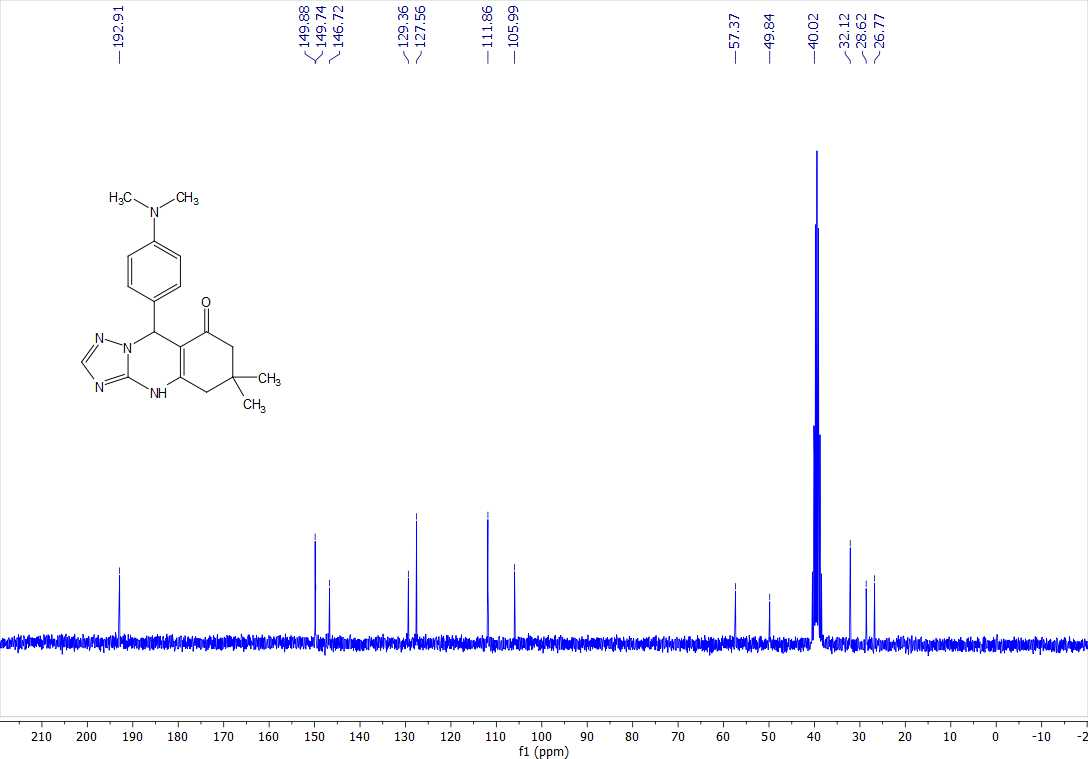


**Scheme 10.** ^1^HNMR and ^13^CNMR for **4j**


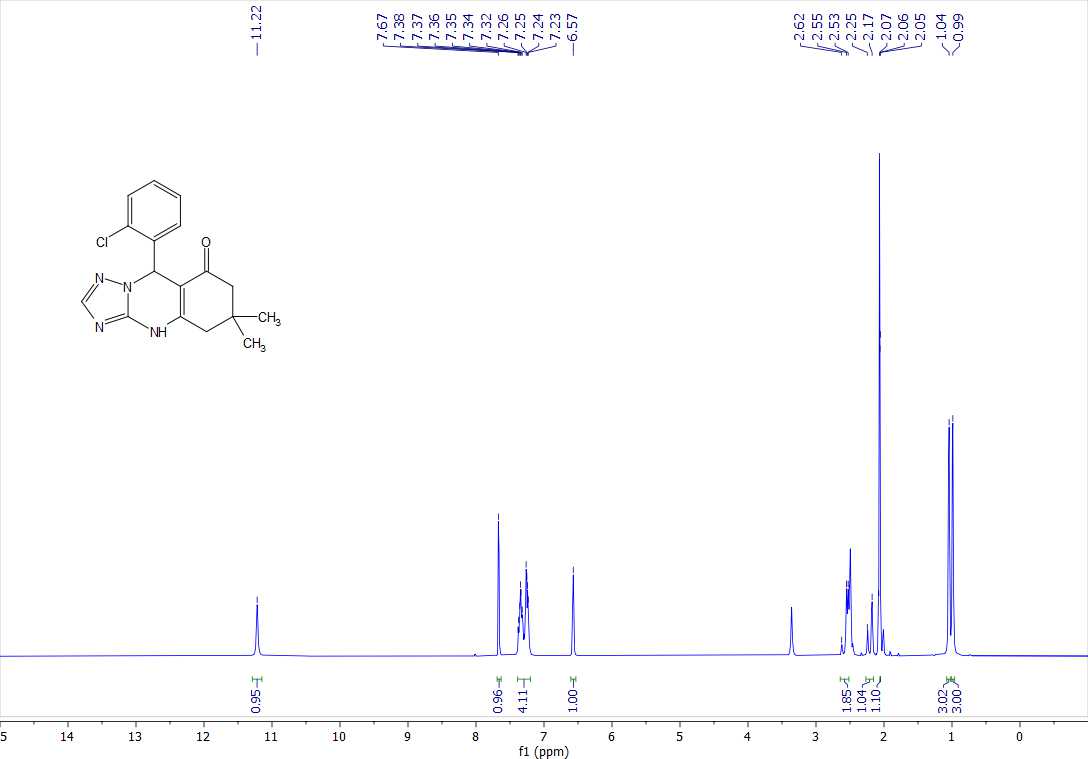


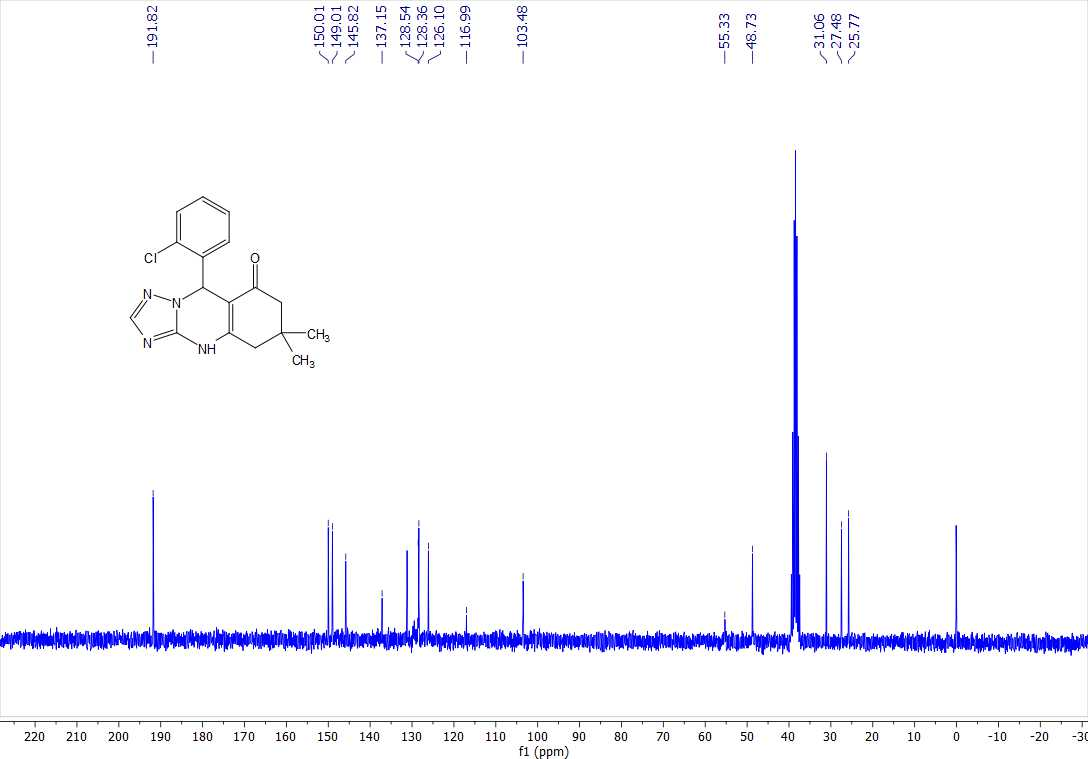


**Scheme 11.** ^1^HNMR and ^13^CNMR for **4k**


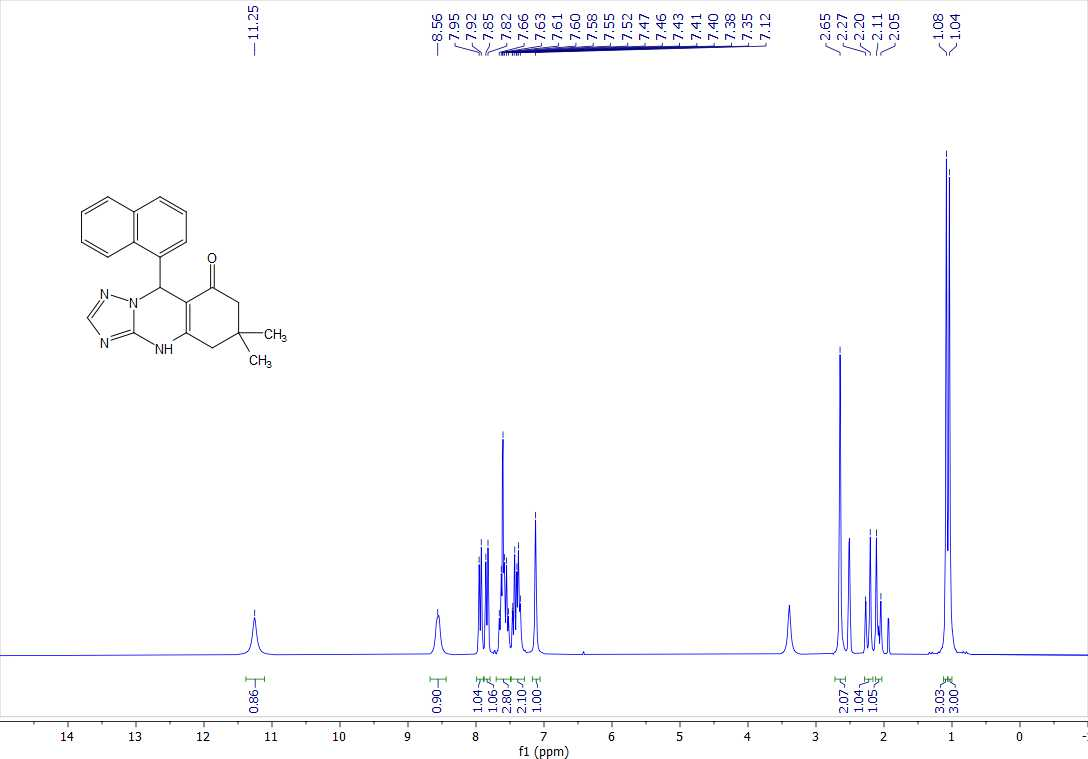


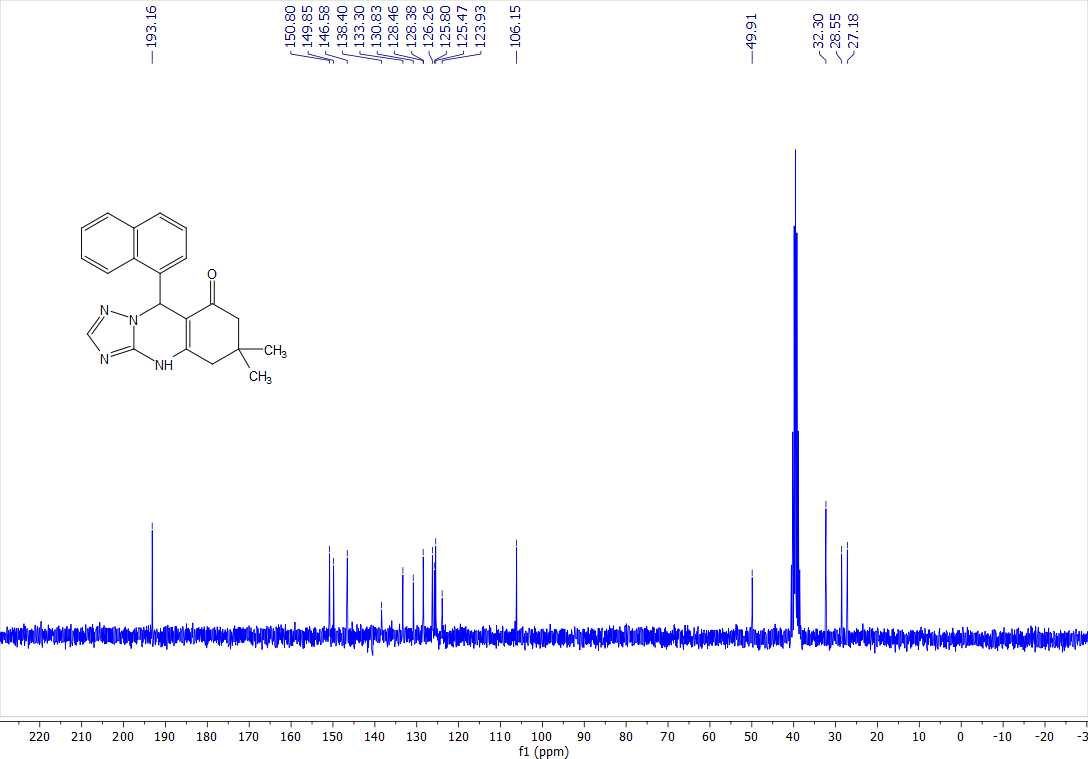


**Scheme 12.** ^1^HNMR and ^13^CNMR for **4l**


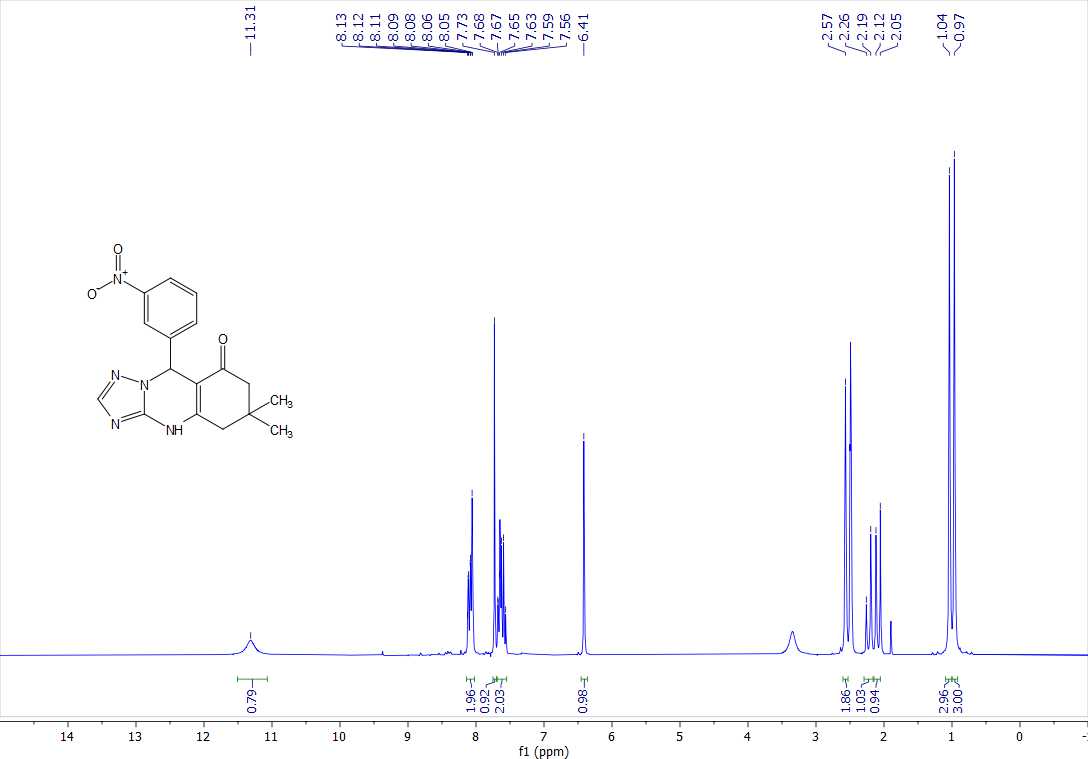


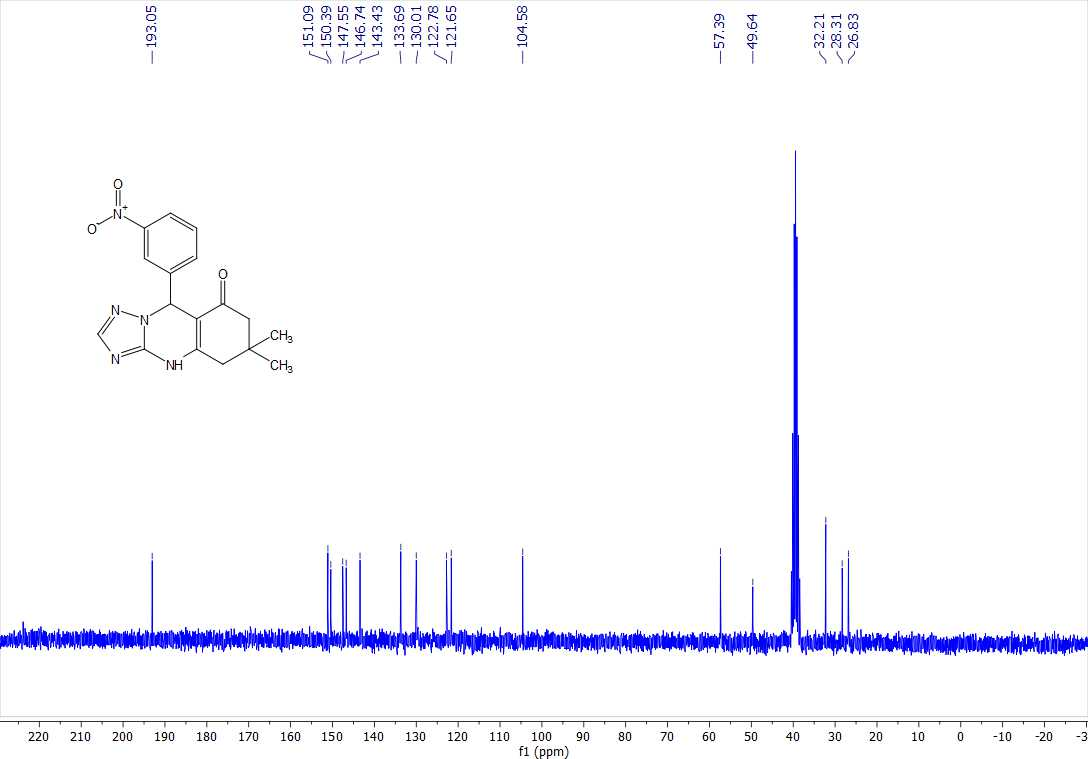


**Scheme 13.** ^1^HNMR and ^13^CNMR for **4m**

**
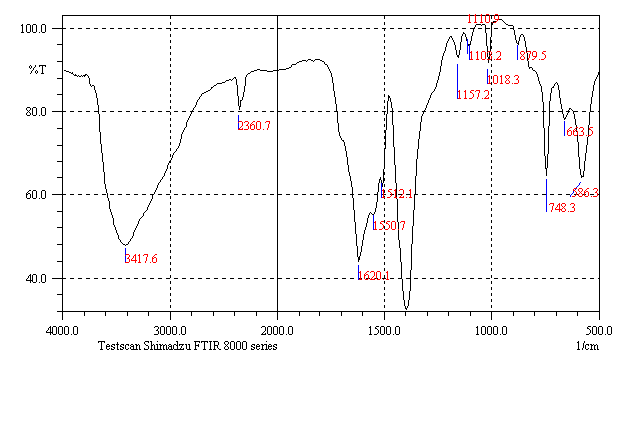
**

**Scheme 14.** FT-IR spectrum of reused MIL-101(Cr) nanocatalyst after eight run.
